# Targeting Sagebrush (*Artemisia* Spp.) Restoration Following Wildfire with Greater Sage-Grouse (Centrocercus Urophasianus) Nest Selection and Survival Models

**DOI:** 10.1007/s00267-022-01649-0

**Published:** 2022-06-10

**Authors:** Cali L. Roth, Shawn T. O’Neil, Peter S. Coates, Mark A. Ricca, David A. Pyke, Cameron L. Aldridge, Julie A. Heinrichs, Shawn P. Espinosa, David J. Delehanty

**Affiliations:** 1 U.S. Geological Survey, Western Ecological Research Center, Dixon Field Station, 800 Business Park Drive, Suite D, Dixon, CA 95620 USA; 2 U.S. Geological Survey, Forest and Rangeland Ecosystem Science Center, 777 NW 9th Street, Suite 400, Corvallis, OR 97330 USA; 3 U.S. Geological Survey, Fort Collins Science Center, 2150 Centre Avenue, Building C, Fort Collins, CO 80526-8118 USA; 4 Natural Resource Ecology Laboratory, in cooperation with U.S. Geological Survey, Fort Collins Science Center, Colorado State University, 2150 Centre Avenue, Building C, Fort Collins, CO 80526-8118 USA; 5 Nevada Department of Wildlife, 6980 Sierra Center Parkway #120, Reno, NV 89511 USA; 6 Department of Biological Sciences, Idaho State University, Pocatello, ID USA

**Keywords:** Cheatgrass, Decision-support tool, Habitat restoration, Nest survival, Sagebrush, Wildfire

## Abstract

Unprecedented conservation efforts for sagebrush (*Artemisia* spp.) ecosystems across the western United States have been catalyzed by risks from escalated wildfire activity that reduces habitat for sagebrush-obligate species such as Greater Sage-Grouse (*Centrocercus urophasianus*). However, post-fire restoration is challenged by spatial variation in ecosystem processes influencing resilience to disturbance and resistance to non-native invasive species, and spatial and temporal lags between slower sagebrush recovery processes and faster demographic responses of sage-grouse to loss of important habitat. Decision-support frameworks that account for these factors can help users strategically apply restoration efforts by predicting short and long-term ecological benefits of actions. Here, we developed a framework that strategically targets burned areas for restoration actions (e.g., seeding or planting sagebrush) that have the greatest potential to positively benefit sage-grouse populations through time. Specifically, we estimated sagebrush recovery following wildfire and risk of non-native annual grass invasion under four scenarios: passive recovery, grazing exclusion, active restoration with seeding, and active restoration with seedling transplants. We then applied spatial predictions of integrated nest site selection and survival models before wildfire, immediately following wildfire, and at 30 and 50 years post-wildfire based on each restoration scenario and measured changes in habitat. Application of this framework coupled with strategic planting designs aimed at developing patches of nesting habitat may help increase operational resilience for fire-impacted sagebrush ecosystems.

## Introduction

The sagebrush biome spans over 630,000 km^2^ of the western United States (Shinneman 2020). Threats to sagebrush ecosystem structure and function encompass altered wildfire regimes (Flannigan et al. 2009), agriculture, energy development (U.S. Fish and Wildlife Service 2015), and anthropogenic impacts such as livestock grazing (i.e., heavy and repeated either constantly or during the growing season; Chambers et al. 2017). Because of disturbances that disrupt key components such as soil stability (Belnap et al. 2009; Eldridge et al. 2016) and trigger changes to vegetation community states (Chambers et al. 2014), there is a need for tools that operationalize theoretical concepts of resilience to disturbance and resistance to non-native invasive plants (hereafter, resilience and resistance; Chambers et al. 2017; Crist et al. 2019; Chambers et al. 2019a). Wildfire is the primary natural disturbance in many ecosystems within the biome, particularly those in the Great Basin (Brooks et al. 2015; Shinneman 2020). Over the past ~30 years, >8.4 mil ha of sagebrush has been burned by wildfires (Brooks et al. 2015; Shinneman 2020), and some areas have burned repeatedly (Brooks et al. 2015; Coates et al. 2016a). The novel grass-fire cycle (D’Antonio and Vitousek 1992) is the genesis of a new, altered regime, whereby non-native invasive annual grasses have thrived with disturbance (Germino et al. 2016) coupled with more weather conditions that favor wildfire and ignition sources (Abatzoglou and Park 2016). The resulting novel feedback cycle has yielded larger and more frequent wildfires (Pilliod et al. 2017; Bradley et al. 2018). Severe wildfire is stand-replacing and effectively removes sagebrush canopy from the landscape through direct post-fire mortality, and recovery is further hampered by potentially limited seed banks and high seed mortality, reduced establishment rates, short seed dispersal distances from surviving plants, and slow growth rate and high mortality among post-fire recruits in suboptimal site conditions (Miller et al. 2013; Shriver et al. 2019; Shinneman 2020).

The underlying sagebrush ecosystem processes driving resilience and resistance of sagebrush communities are becoming well-understood (Chambers et al. 2014; Maestas et al. 2016; Chambers et al. 2019a). Variation in resilience and resistance and associated productivity align with elevation and soil moisture gradients, whereby low elevation sites with warm, dry soil composed largely of Wyoming big sagebrush (*Artemisia tridentata* ssp. *wyomingensis*) are prone to state transitions and difficult to restore. In contrast, resilient and readily restorable sites exist more frequently at higher elevations with cool, moist soils dominated by mountain big sagebrush (*A. t*. ssp. *vaseyana*). The recent advent of spatially explicit maps depicting soil climate regimes and related ecological site potential has greatly operationalized resilience and resistance concepts and facilitated understanding of where, when, and how to implement post-wildfire restoration across sagebrush ecosystems in the western United States (Maestas et al. 2016; Chambers et al. 2017; Chambers et al. 2019b). Such resilience-based information facilitates tools that predict and assess outcomes of passive and active restoration actions (Suding 2011; Chambers et al. 2019a).

Nevertheless, ecological metrics with broad spatial extents like resilience and resistance are based on gradual processes that influence plant community characteristics (Chambers et al. 2014), and may be out of sync with generation times or life history requirements of wildlife species intended to benefit from restoration of their habitat (Miller and Hobbs 2007; Perring et al. 2015; Ricca and Coates 2020), rendering many habitat restoration efforts ineffective (Miller and Hobbs 2007). The potential for such spatial and temporal mismatches is a concern for managing Greater Sage-Grouse (*Centrocercus urophasianus*; hereafter, sage-grouse), which is a sagebrush-obligate species considered for listing multiple times under the Endangered Species Act (U.S. Fish and Wildlife Service 2015) and is at the nexus of 21st century sagebrush conservation planning efforts (Bureau of Land Management 2020). Sage-grouse rely on a diversity of vegetation communities within sagebrush ecosystems to fulfill habitat needs across their life history stages, while its distribution also encompasses habitat for several other sagebrush-obligate species (Rowland et al. 2006; Hanser and Knick 2011; Knick and Connelly 2011; Coates et al. 2016b). Although sage-grouse populations are cyclical (Row and Fedy 2017), largely in response to variable climatic conditions (Coates et al. 2020), prevailing long-term trends have exhibited downward trajectories across much of the species’ range during recent decades (Connelly et al. 2004; Garton et al. 2011; Coates et al. 2021). Wildfire, followed by vegetation type conversion to non-native annual grasslands, is thought to be a primary driver of sage-grouse declines across the Great Basin (Connelly et al. 2011; Coates et al. 2016a). Although sensitivity of different life stages to wildfire impacts has not been thoroughly studied, recent evidence suggests that the loss of shrubs from wildfire has a substantial impact on nest survival of sage-grouse (Lockyer et al. 2015; Foster et al. 2018; O’Neil et al. 2020). Sage-grouse rely on shrub canopy cover for nesting concealment and thermal refugia (Connelly et al. 2004; Coates et al. 2017), and loss or slow recovery of this critical shrub cover following wildfire reduces nest habitat quality (Beck et al. 2009). Reductions in nest survival can limit recruitment and may reduce population growth rates (Taylor et al. 2012; Foster et al. 2018; Dudley et al. 2021). Furthermore, hen fidelity to pre-fire nest sites may reduce survival because sites that previously comprised high quality habitat lack shrub and other habitat components after wildfire (Lockyer et al. 2015; Foster et al. 2018; O’Neil et al. 2020). Thus, for post-fire habitat restoration to be effective for species such as sage-grouse, the relatively short-term requirements of the species warrant consideration when allocating resources to proposed restoration sites and treatment options that may take longer to yield desired outcomes.

Additionally, targeted resource allocation is important because: the sheer area of sage-grouse habitat burned annually (1.3 million ha from 2015–2017; US Department of the Interior 2017) presents challenges for practical application of restoration, including increased resource demand (Copeland et al. 2018); post-fire sagebrush establishment has proven difficult due to variation in restoration practices (e.g., seed source, planting method, timing, logistics) and underlying resilience and resistance or ecological site potential (Meinke et al. 2009; Arkle et al. 2014; Pyke et al. 2015); availability and predictability of weather favorable to post-wildfire recovery contributes additional uncertainty to potential for treatment success (Shriver et al. 2018; O’Connor et al. 2020); and transient demographic and population dynamics of sagebrush yield lower canopy cover, where post-disturbance sagebrush populations have greatly altered vital rates and size structure from pre-disturbance populations (Shriver et al. 2019). To address these challenges, decision-support frameworks for post-fire restoration can become more operational through explicit identification of site qualities that were selected by nesting sage-grouse and yielded high nest survival (i.e., positive fitness consequences) before wildfire, and further understanding which site characteristics are extrinsic to wildfire and which will be degraded or altered by wildfire. Additionally, these quantitative frameworks can generate spatial predictions of immediate and longer-term changes to nesting habitat quality (i.e., selection choice plus fitness consequence) given underlying resilience and resistance and planned restoration practices. When applicable, these frameworks can incorporate animal behavior that influences habitat selection to better target management actions (Connelly et al. 2011; Doherty et al. 2016). For example, sage-grouse have a propensity to select nest sites adjacent to leks (Holloran and Anderson 2005; Coates et al. 2013). Parameterizing habitat suitability models to include this behavior refines predicted habitat delineations (Ricca et al. 2018; Ricca and Coates 2020). This framework, when replicated, would facilitate creation of resource islands, or strategic and concentrated plantings of sagebrush (Hulvey et al. 2017), in areas where sage-grouse would be predicted to nest and thus where sagebrush restoration would potentially succeed. Such a response would thereby increase synchrony between and sagebrush recovery times and sage-grouse post-fire population dynamics (Ricca and Coates 2020).

Herein, we describe a framework for targeted post-fire restoration using predictive models of sage-grouse habitat quality as defined by areas selected for nesting with high nest survival which are then integrated with underlying ecological capacity for sagebrush to recover with different restoration practices following wildfire. Our framework incorporates sage-grouse behavior, namely the use of productive habitats adjacent to leks, to improve predictions of high productivity nesting areas that reinforce prioritization of restoration efforts. We used an expansive radio telemetry dataset at a long-term monitoring site in northwestern Nevada to model sage-grouse nest site selection and survival during pre-fire conditions, and then predicted expected differences on the same landscape following two major wildfires resulting in an immediate and acute loss of sagebrush. Differences among predicted surfaces formed the basis of a habitat restoration index for sage-grouse. We projected future conditions following active or passive restoration efforts by incorporating spatially explicit sagebrush community recovery rates and risk of annual grass dominance that varied with resilience and resistance under three scenarios: passive recovery, seeding, and transplanting sagebrush seedlings. This identified areas that could be prioritized for restoration given a modeled return to a minimum of 20% sagebrush cover as required by nesting sage-grouse (Connelly et al. 2004; Smith et al. 2020) within 30 or 50 years. This quantitative framework facilitates holistically targeting areas for post-fire restoration at scales meaningful for management by reducing spatial and temporal mismatches arising from longer sagebrush ecosystem recovery times and more immediate sage-grouse population responses. Supporting data are provided via USGS ScienceBase repository (10.5066/P96K6X05; Roth et al. 2022)

## Materials and Methods

### Study Area

Our study area was the Virginia Mountains of northwestern Nevada, USA. The Virginia Mountains occur in the northwestern Great Basin, a high desert (>2000 m) characterized by sagebrush shrub steppe with relatively low annual precipitation occurring mainly as snow and primarily used as rangeland. Vegetation was dominated by low sagebrush (*A. arbuscula*), and mountain big sagebrush (*A. t. vaseyana*) at higher elevations, and Wyoming big sagebrush (*A. t. wyomingensis*) and black sagebrush (*A. nova*) occurring at lower elevations. Non-sagebrush shrubs included rabbitbrush (*Chrysothamnus* ssp.), Mormon tea (*Ephedra viridis*), snowberry (*Symphoricarpos* ssp.), western serviceberry (*Amelanchier alnifolia*), and antelope bitterbrush (*Purshia tridentata*). Conifer woodlands were present but not prevalent, comprising single-leaf pinyon pine (*Pinus monophylla*) and Utah juniper (*Juniperus osteosperma*) (hereinafter, both referred to as “pinyon-juniper”). Non-native invasive annual grasses included cheatgrass (*Bromus tectorum*) and medusahead (*Taeniatherum caput-medusae*) (hereinafter, both referred to as “non-native invasive annual grasses”). Native perennial grasses included needle and thread (*Hesperostipa comata*), Indian ricegrass (*Achnatherum hymenoides*), bluebunch wheatgrass (*Pseudoroegneria spicata*), Sandberg bluegrass (*Poa secunda*), and squirreltail (*Elymus elymoides*).

Our analysis centered on two large wildfires that impacted the study area during the summers of 2016 and 2017. In August 2016, the Virginia Mountains Complex Fire burned a total of 24,171 hectares. During July of 2017, the study area was impacted by the Long Valley Fire that burned a total of 33,886 hectares (https://inciweb.nwcg.gov/incident/5354/). The fires were fueled by grass and sagebrush and occurred within the core distribution of local sage-grouse populations, affecting 48.5% of breeding and nesting habitat. Intersection of nest site locations from sage-grouse data collected between 2009–2016 (see Data Collection and Materials) and wildfire perimeter data revealed that 60% of sage-grouse nest locations previously occurred in the affected area.

### Data Collection and Materials

We identified and monitored sage-grouse nest locations during the breeding seasons of 2009–2016, prior to the wildfires. We used spotlighting techniques (Giesen et al. 1982; Wakkinen et al. 1992) to capture female sage-grouse. Most sage-grouse were outfitted with necklace-style VHF radio-transmitters (Kolada et al. 2009), while a subsample were fitted with combined Global Positioning Systems (GPS) - Platform Transmitter Terminals (North Star Science and Technology, LLC, King George, Virginia) and micro VHF transmitters during 2012–2016. We relocated sage-grouse at a minimum of twice weekly during the nesting season (March–June) to identify nests and determine nest fates (O’Neil et al. 2020). Nest locations were documented using handheld GPS and subsequently monitored until nest fate was determined by examination of nest bowl remains, where a successful nest was indicated by the presence of ≥1 hatched egg or chick in the nest bowl.

### Landscape Covariates

We used Geographic Information Systems to quantify a suite of landscape metrics that could potentially influence sage-grouse nest site selection and nest success. Landscape predictors broadly covered variation in surface cover of dominant vegetation (30 × 30 m; Xian et al. 2015), topography, classification of regional soil moisture and temperature annual ranges, hydrography, and anthropogenic infrastructure. We summarized spatial predictors across multiple scales relevant to sage-grouse nesting and movement (i.e., mean percent cover of annual grass; 30 × 30 m) and we calculated distance metrics to relevant features (i.e., nearest distance to road). We conducted neighborhood spatial analyses within ArcMap 10.3 (ESRI, Redlands, CA, USA). A complete list of landscape metrics and their data sources is provided in Supplementary Table S1 in the supplementary material. Because we initially considered many candidate predictors across multiple spatial scales and distance representations, it was necessary to reduce the size of this dataset prior to fitting final models of nest selection and survival. We did this by selecting only the most influential predictors from sets of correlated predictors (e.g., multiple scales) with an iterative model subset routine, where the contributions of each predictor to model fit (via *d*AIC rankings) were assessed independently of those that were correlated. This variable reduction procedure is described fully in Supplementary Material S2.

### Pre-Fire Nest Site Selection Model

We quantified patterns of nest site selection with a resource selection function (RSF) study design (Johnson et al. 2006), where habitat characteristics were contrasted between used locations (nests) and random (available) locations occurring within 17 km of known, active leks. This distance constraint was applied because nests are known to occur near leks (Coates et al. 2013), and 17 km was the maximum distance observed in the dataset (O’Neil et al. 2020). As such, nest site availability represented a range of site conditions encompassing the most probable distribution of nesting locations, given their proximity to known lekking grounds. We generated ten random locations per nest within the buffered area around active leks for the Virginia Mountains sub-population. The choice of a 10:1 ratio of random to used locations was intended to appropriately weight the available distribution (e.g., maximizing the number of available locations; Northrup et al. 2013; Fieberg et al. 2021) without oversaturating these locations within the study’s geographic extent. We used a Bayesian generalized linear mixed model (GLMM), and allowed separate intercepts for each individual sage-grouse and year, to help account for the unbalanced sampling efforts as well as intraclass correlation among repeated measures and samples collected within the same year (Gillies et al. 2006). The response variable was binary for nest location (1) or random location (0), so we assumed a binomial error distribution when fitting the model. We selected candidate habitat predictors based on the results of variable reduction methods described in Supplementary Material S2 and standardized each variable prior to model fitting (mean = 0, sd = 1).

We obtained estimates for the nest RSF GLMM habitat variables using MCMC methods in JAGS 4.2.0 (Plummer 2003), accessed via R with *rjags* (Plummer 2018) and *jagsUI* (Kellner 2018). We specified shrinkage prior distributions for habitat covariates using the Bayesian lasso (Park and Casella 2008; Supplementary Material S3), and uninformative normally distributed prior distributions for random effects (Supplementary Material S3). Additional details about the model process and MCMC parameter settings are available in Supplementary Material S3. We reported the results of all coefficient estimates (i.e., fixed effects) in terms of median posterior values and 2.5th and 97.5th percentiles (95% CRI).

### Pre-Fire Nest Survival Model

We used a Bayesian shared frailty model (Halstead et al. 2012) to model nest success, where daily nest hazard risk was a function of landscape habitat covariates, age of hen, and day of season. The frailty model accounts for the nest exposure period by treating each day in the available nest encounter history as a Bernoulli trial, where each nest either survived or did not. Encounter histories for each nest consisted of known active and censored days throughout the nesting season, ending with either a successful or failed nest (e.g., Converse et al. 2013). Nests that ultimately failed were assumed to have failed between their last active date (e.g., last checked) and the date that failure was determined. As described for the nest RSF, we selected candidate habitat predictors based on the variable reduction method (Supplementary Material S2), applied the Bayesian lasso priors to the model’s habitat coefficients, and incorporated random intercepts for year and individual. Results from this model were converted from daily unit hazard to a 38-day cumulative nest survival estimate for mapping purposes. The frailty model was also fit using MCMC in JAGS. Model specification and parameter settings are described in detail in Supplementary Material S4.

### Post-Fire Habitat Suitability Modeling

To identify the highest priority areas for potential sagebrush restoration, we used the final nest site RSF and nest frailty model to generate predicted surfaces of relative nest selection and probability of nest success across the study area. We did this by applying model coefficients from the fitted model equations (median estimates from posterior distributions) to their associated habitat predictor values at each 30 m raster pixel, and scaling to a pre-fire RSF and a 38-day survival estimate for each model, respectively. Notably, the distance to lek predictor was included in these predictions to account for clustering and the declining probability of birds using nesting habitat as distance away from breeding grounds increases (Holloran and Anderson 2005; Coates et al. 2013; Doherty et al. 2016), thereby preventing direction of restorative efforts to areas not used by sage-grouse. Detailed methods used to map relative nest selection and nest success are available in Supplementary Materials S3 and S4. These surfaces represented habitat quality potential and contributions to nesting productivity prior to the wildfires.

We then generated new predicted surfaces of relative nest selection and probability of nest success by applying the original, pre-fire model coefficients to the covariates in a simulated post-fire landscape. We simulated the immediate loss of habitat by replacing mapped values of shrubland habitat covariates with 0, which assumed complete loss of shrub cover immediately following wildfire. Values for annual grass and NDVI were updated to post-fire conditions using a recently developed geospatial layer (Boyte and Wylie 2018). To estimate the potential cover of perennial herbaceous vegetation immediately (<5 years) after wildfire, we used the geospatial layer from Maestas et al. (2016) depicting ecosystem resilience to disturbance and resistance to invasion (i.e., classes high, moderate, and low) to index ecological productivity and update post-fire values. Due to data limitations on grazing regimes across our site, we were unable to directly quantify the potential for post-fire grazing to delay the recovery of perennial grasses or to increase the colonization of non-native annual grasses. However, we did include perennial grass recovery in our post-wildfire landscape. In burned pixels classified as either the moderate or high resilience and resistance class, we allowed perennial cover to return to 50 and 75% of its initial value, respectively. In less productive burned pixels classified as the low resilience and resistance class, we allowed perennial cover to return to 25% of its initial value (Miller et al. 2013).

We scaled the post-fire RSF between 0 and 1 relative to the minimum and maximum values of the pre-fire RSF (e.g., Ricca et al. 2018) as described in Supplementary Material S3. To develop an index representing loss of habitat within burned areas, we first subtracted the post-fire RSF from the pre-fire RSF to obtain the difference in nest RSF values (*Δ*RSF) associated with the fires. Similarly, we subtracted post-fire nest survival predictions from pre-fire estimates to obtain the difference in expected nest survival contributions (*Δ*S). We identified the most suitable areas for restoration planting activities based on the combination of *Δ*RSF and *Δ*S. To delineate this area for prioritization of restoration, we first converted each surface into binary values based on their 50th percentiles (i.e., 0 < 50th percentile, 1 > 50th percentile), and then combined the two surfaces to represent a ranked restoration index class: 1 = low *Δ*RSF, low *Δ*S (0,0); 2 = low *Δ*RSF, high *Δ*S (0,1); 3 = high *Δ*RSF, low *Δ*S (1,0), 4 = high *Δ*RSF, high *Δ*S (1,1). The 50th percentile was chosen in part because it occurred at the value of ~ 0 for both *Δ*RSF and *Δ*S, so the highest quality ranking could be interpreted as areas that lost habitat from fire based on both selection and survival model contributions. Our percentile-based approach for combining selection and survival probabilities follows Aldridge and Boyce 2007. We subsequently prioritized restoration suitability in class 3 and 4, because they represented the immediate loss of areas that were previously highly selected and targeted areas with the greatest loss in nest survivorship (in the case of restoration class index 4). Sage-grouse continue to use habitat following wildfire (O’Neil et al. 2020; Dudley et al. 2021), so prioritizing high selection, low survival areas can help ameliorate potential post-wildfire ecological traps.

### Post-Fire Habitat Recovery Modeling

We evaluated the expected success of habitat recovery in priority areas after 30 and 50 years for four different restoration scenarios: (1) passive recovery; and post-wildfire restoration through (2) grazing exclusion, (3) seeding, or (4) transplanting sagebrush. We used the LANDFIRE biophysical settings raster (Rollins 2009) to map the extent of three different sagebrush communities within the study area (Table 1). The biophysical settings raster categorizes the landscape into potential dominant vegetation systems by applying expert-based state and transition models assuming pre-European settlement disturbance regimes (e.g., fire return intervals) to the current biological and physical site conditions. We classified the sagebrush biophysical settings into three major sagebrush communities: Wyoming big sagebrush (*Artemisia tridentata wyomingensis, A.t. tridentata)*; mountain big sagebrush (*A. t. vaseyana)*; and low sagebrush (*A. nova, A. arbuscula) (*Brooks et al. 2015). It is well-established that interactions between elevation and underlying soil moisture/temperature gradients mediate plant communities’ resilience and resistance (Chambers et al. 2014; Chambers et al. 2017). Sagebrush communities occurring at lower elevations (<2000 m) within mesic/aridic (warm and dry) zones face non-optimal growing conditions for native perennials as well as potential competition from non-native annuals and therefore are less likely to recover at their estimated maximum rate than those in frigid/xeric (cool and moist), higher elevation conditions (Miller et al. 2013). We modified annual recovery rates based on their occurrence within high, moderate, or low resilience and resistance classes (Table 1). For this analysis, we calculated annual recovery rates by dividing 20% sagebrush cover, which is the minimum amount of cover needed to fulfill sage-grouse life history requirements (Connelly et al. 2004; Coates et al. 2017; Smith et al. 2020), by the number of years required to achieve that amount of cover (Table 1). Recovery rates were determined for each sagebrush community by resilience and resistance class from a review of existing literature describing post-fire growth rates and soil and elevation characteristics. While there is a relative wealth of empirical information for mountain big sagebrush (Baker 2006; Ziegenhagen and Miller 2009; Baker 2011; Miller et al. 2013; Nelson et al. 2014) and Wyoming big sagebrush (Nelle et al. 2000; Baker 2006; Cooper et al. 2011; Arkle et al. 2014; Bates Jonathan et al. 2020) recovery, there was a paucity of information for low sagebrush recovery. Given its position along the elevational/productivity gradient (Miller et al. 2013), we assigned an annual recovery rate that was the average of mountain big and Wyoming big sagebrush. This approach yielded the highest recovery potential for mountain sagebrush in the high resilience and resistance class and lowest recovery potential for Wyoming big sagebrush communities in the low resilience and resistance class. Because disturbance occurring in the low resilience and resistance class is expected to lead to dominance by non-native annual grass (Chambers et al. 2017; Pilliod et al. 2017), we further randomly converted 90% of recoverable pixels in this class to annual grass. We included a lag year in the recovery rates for all classes to represent time required for germination. Because sagebrush recovery is highly dependent on germination and early survivorship post-disturbance (Dettweiler-Robinson et al. 2013; McAdoo et al. 2013; Pyke et al. 2020), we accounted for reduced establishment rates caused by wildfire damage to the seedbank by randomly assigning a recruitment probability from a uniform distribution between 10–25% to each pixel containing sagebrush within the burn scars, assuming adequate seed depths to allow viability, emergence and survival (Wijayratne and Pyke 2012). Pixels that were recruited recovered based on the annual rates established for their community and resilience and resistance class.

**Table 1:** Recovery times for the different sagebrush communities that occur within the Virginia Mountains study site within Nevada, USA

Scenario	Probability of establishment	Community	Resilience and resistance class	Recovery (years)	Sources
Passive	10–25%	Wyoming	High	50	Moffet et al. 2015; Nelson et al. 2014; Miller et al. 2013, Cooper et al. 2011, Lesica et al. 2007, Baker 2006, Colket 2003, Wambolt et al. 2001, Watts and Wambolt (1996)
			Moderate	123	
			Low	246	
		Low	High	22	Miller et al. 2013
			Moderate	38	
			Low	76	
		Mountain big	High	9	Bates Jonathan et al. 2020; Shinneman and McIlroy 2016; Miller et al. 2013, Lesica et al. 2007, Ziegenhagen and Miller 2009, Baker 2006
			Moderate	15	
			Low	30	
Grazing Exclusion	15–30%	Wyoming	High	50	Moffet et al. 2015; Nelson et al. 2014; Miller et al. 2013, Cooper et al. 2011, Lesica et al. 2007, Baker 2006, Colket 2003, Wambolt et al. 2001, Watts and Wambolt (1996)
			Moderate	123	
			Low	246	
		Low	High	22	Miller et al. 2013
			Moderate	38	
			Low	76	
		Mountain big	High	9	Bates Jonathan et al. 2020; Shinneman and McIlroy 2016; Miller et al. 2013, Lesica et al. 2007, Ziegenhagen and Miller 2009, Baker 2006
			Moderate	15	
			Low	30	
Seeding	25–50%	Wyoming	High	49	Dettweiler-Robinson et al. 2013, McAdoo et al. 2013, Pyke et al 2020.
			Moderate	122	
			Low	244	
		Low	High	21	
			Moderate	37	
			Low	74	
		Mountain big	High	8	
			Moderate	14	
			Low	28	
Seedling transplant (1 seedling/ 1 m^2^)	50–100%	Wyoming	High	21	Dettweiler-Robinson et al. 2013, McAdoo et al. 2013, Pyke et al 2020.
			oderate	77	
			Low	144	
		Low	High	14	
			Moderate	24	
			Low	48	
		Mountain big	High	6	
			Moderate	10	
			Low	20	

Recovery rates were modified by the resilience and resistance class in which the sagebrush community occurred. Additionally, growth rates were projected under four different management scenarios: passive, grazing exclusion, seeding, and seedling transplant. Annual recovery rates were calculated based on the number of years necessary for each sagebrush community to achieve 20% cover, which is required for sage-grouse nesting. Passive and seeding scenarios include an additional lag year to account for germination time. While annual recovery rates were identical in the passive and grazing exclusion scenario, grazing exclusion scenario had a higher rate of establishment. The seeding and seedling transplant scenarios also had higher rates of establishment than the passive scenario.

We simulated recovery for the three restoration scenarios by either modifying passive recruitment probability or both recruitment probability and annual recovery rates. We developed a restoration scenario that simulated sagebrush recovery under grazing exclusion. Because livestock grazing is so pervasive throughout sagebrush ecosystems (Williamson et al. 2020), we assume our literature-derived recovery rates for the baseline passive scenario are impacted by livestock grazing pressure. Livestock disperse annual grass seeds, and grazing reduces native herbaceous cover while trampling disturbs biological soil crusts that typically fill interspace and prevent invasion by annual grass (Reisner et al. 2013; Ellsworth et al. 2016; Condon and Pyke 2018; Williamson et al. 2020). The grazing exclusion scenario assumed an increased probability of sagebrush establishment promoted by reduced competition from annual grass (Reisner et al. 2013; Ellsworth et al. 2016; Condon and Pyke 2018) and soil disturbance. We simulated this effect by increasing the range of recruitment probability to 15–30%, which still accounts for the independent negative impact of wildfire on annual grass colonization (Condon and Pyke 2018; Williamson et al. 2020) and sagebrush seedbank depletion (Baker 2011; Wijayratne and Pyke 2012). Because livestock generally do not graze on sagebrush, we assumed livestock exclusion would not increase observed cover compared to the passive scenario, so we maintained the passive annual recovery rates.

For seeding and seedling transplant, we altered both the probability of establishment as well as annual recovery rates. We increased the range of recruitment probability to 25–50% to simulate increased potential for establishment due to seeding (Pyke et al. 2020). Additionally, we doubled the initial recovery rate for second year seeding treatments to simulate increased germination during the growing season immediately following treatment. For restoration using transplanted seedlings, we again doubled the recruitment probability to 50–100%, as this method eliminates variability in survivorship due to unsuccessful germination and establishment and is only impacted by early survivorship (Dettweiler-Robinson et al. 2013; Pyke et al. 2020). Accordingly, we doubled recovery in the first year to represent establishment, and then set recovery at 1.5 times the passive annual rate in subsequent years to account for increased cover. For each restoration scenario, we projected the annual recovery over 30 and 50 years to produce binary rasters identifying areas with > = 20% cover. We intersected recovered areas from each restoration scenario with restoration index classes 3 and 4 to identify post-fire areas with the highest sagebrush restoration potential and recovery of formerly quality nesting habitat for sage-grouse. We provide code for generating annual percent cover recovery maps in the three sagebrush communities for each recovery scenario in Supplementary Materials S5.

## Results

### Nest Site Selection

**Fig. 1:**
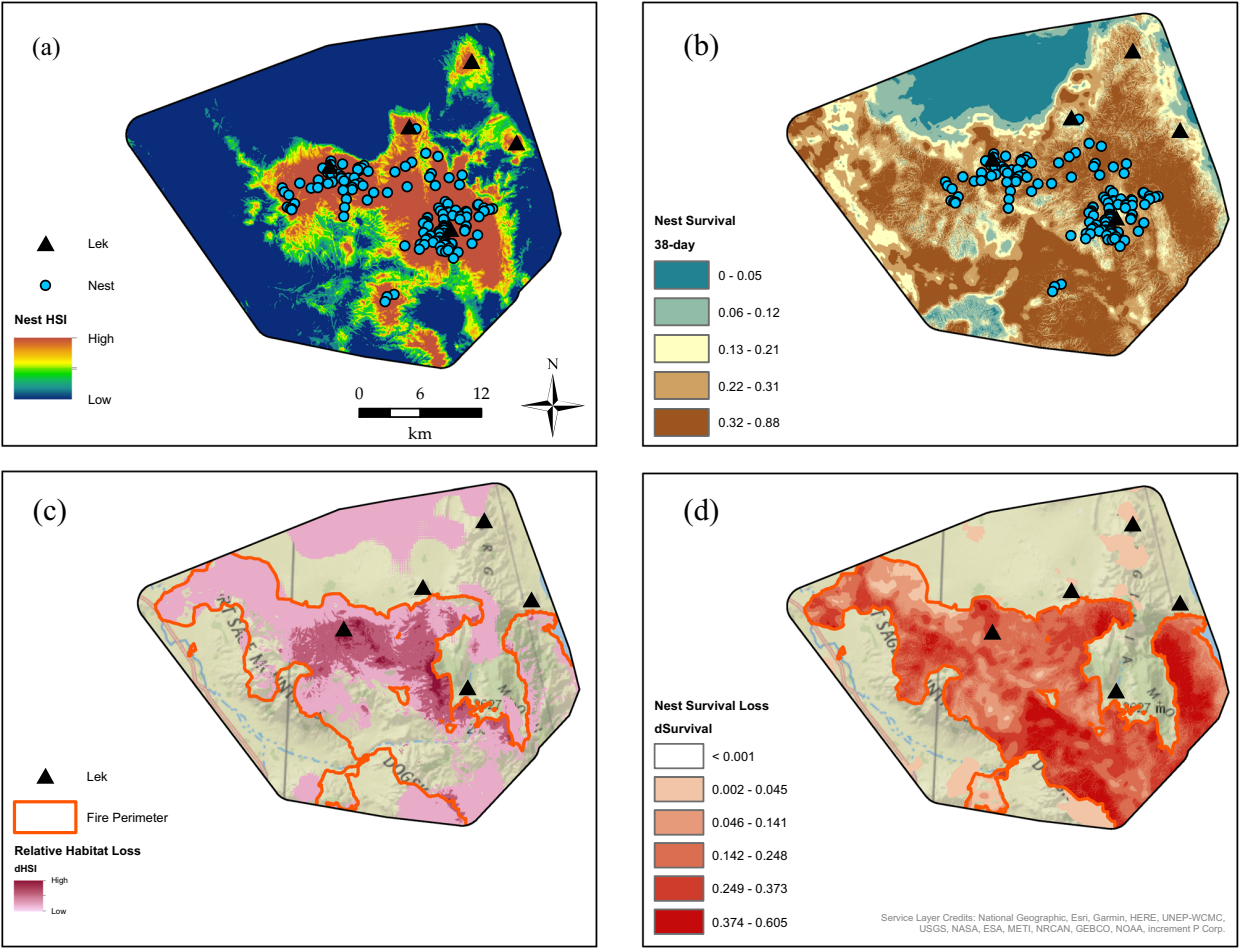
Predictions of (**a**) pre-fire sage-grouse nest habitat suitability index (HSI); (**b**) pre-fire sage-grouse nest survival map; (**c**) projected loss of selected nesting habitat from wildfire; (**d**) projected loss of habitat contributions to nest survival from wildfire within the Virginia Mountains region of Nevada, USA. Pre-fire data were specific to the breeding seasons of study years 2009–2016. Wildfire events occurred during the summers of 2016 and 2017.

Prior to the wildfires of 2016 and 2017, we located and monitored 141 nests from 96 sage-grouse over 8 years (2009–2016). Of the 141 nests, 71 had hatched eggs (50.3%). After accounting for exposure time via the nest frailty model, the pre-fire median posterior estimate of cumulative 38-day nest survival was 0.336 (95% CRI = 0.141–0.577). Of all nests, 84 (59.6%) occurred within the area that eventually burned. Sage-grouse in the Virginia Mountains study region selected nest sites where proportions of big sagebrush cover (radii [*r*] = 75 m; Table 2) and perennial herbaceous cover (*r* = 439 m; Table 2) were above average compared to available locations, and where percentages of pinyon-juniper cover class 1 (1–10% cover; *r* = 1451 m) and bare ground (*r* = 439 m) were below average (Table 2). With respect to topography, nest sites occurred where surface curvature values (*r* = 1451) were above average (more concave; Table 2). Nest site locations also occurred closer to perennial streams and where intermittent stream densities (*r* = 75 m; Table 2) were above average compared to available locations, and where negative anthropogenic effects included proximity to primary or secondary road (avoided, relative to available locations; Table 2). Nest site selection increased strongly with proximity to active leks (Table 2). The distribution of selected nesting resources predicted across the predicted pre-fire study area is shown in Fig. 1. Posterior distributions of model coefficients are shown in Supplementary Materials S6 (Figs. S6–1).

**Table 2:** Posterior distribution estimates of habitat predictor covariates from a model of nest site selection (nest resource selection function, or nest RSF) in the Virginia Mountains region of Nevada, USA

Habitat predictor	Scale	$\widehat \beta$ (50th)	2.5th	97.5th	*pd*	Influence	Source
% Cover big sagebrush	*r* = 75 m	0.737	0.382	1.116	1.000	+	https://www.mrlc.gov/data
Curvature	*r* = 1451 m	0.751	0.465	1.053	1.000	+	https://viewer.nationalmap.gov/basic/
							https://evansmurphy.wixsite.com/evansspatial/arcgis-gradient-metrics-
Active lek	Exp. Decay^a^	6.001	4.234	7.959	1.000	+	original data collection
Perennial stream	Exp. Decay^a^	3.155	1.351	5.053	1.000	+	https://www.usgs.gov/core-science-systems/ngp/national-hydrography
% Cover perennial herbaceous	*r* = 439 m	0.873	0.308	1.482	0.999	+	https://www.mrlc.gov/data
% Cover pinyon-juniper (<10%)	*r* = 1451 m	−0.953	−1.742	−0.253	0.997	−	https://www.sciencebase.gov/catalog/item/59160b60e4b044b359e32e67
Primary/secondary road	Exp. Decay^a^	−4.962	−0.079	−0.756	0.995	−	https://www.census.gov/geo/maps-data/data/tiger-line.html
% Cover bare ground	*r* = 439 m	−0.930	−1.675	−0.203	0.993	−	https://www.mrlc.gov/data
Intermittent stream density	*r* = 75 m	0.293	0.039	0.559	0.988	+	https://www.usgs.gov/core-science-systems/ngp/national-hydrography
High-voltage power line	Exp. Decay^a^	1.976	0.170	4.012	0.987	+	https://www.spglobal.com/platts/en/products-services/electric-power
Compound topographic index	*r* = 1451 m	−1.100	−2.430	0.021	0.972	−	https://viewer.nationalmap.gov/basic/
							https://evansmurphy.wixsite.com/evansspatial/arcgis-gradient-metrics-
% Cover other sagebrush	*r* = 439 m	−0.483	−1.006	0.010	0.972	−	https://www.mrlc.gov/data
% Agriculture	*r* = 1451 m	−1.276	−4.316	0.041	0.969	−	https://www.landfire.gov/
Spring	Exp. Decay^a^	1.129	−0.161	2.665	0.950	+	https://www.usgs.gov/core-science-systems/ngp/national-hydrography
Normalized difference vegetation index	*r* = 1451 m	0.295	−0.064	0.685	0.945	+	10.5067/MODIS/MOD13Q1.0062015
% Cover annual grass	*r* = 1451 m	0.285	−0.079	0.687	0.936	+	https://www.mrlc.gov/data
Transformed aspect	*r* = 75 m	−0.178	−0.517	0.129	0.863	−	https://viewer.nationalmap.gov/basic/
Agricultural field	Exp. Decay^a^	−0.465	−2.502	1.074	0.728	−	https://www.landfire.gov/
Topographic roughness	*r* = 1451 m	−0.118	−0.557	0.272	0.724	-	https://viewer.nationalmap.gov/basic/
							https://evansmurphy.wixsite.com/evansspatial/arcgis-gradient-metrics-
Non-sagebrush shrub	*r* = 75 m	−0.070	−0.374	0.223	0.684	−	https://www.mrlc.gov/data
Road density	*r* = 1451 m	−0.110	−0.634	0.367	0.682	−	https://www.census.gov/geo/maps-data/data/tiger-line.html
Intermittent stream density	*r* = 1451 m	0.025	−0.573	0.601	0.536	+	https://www.usgs.gov/core-science-systems/ngp/national-hydrography

Data were specific to the breeding seasons of study years 2009–2016, prior to significant wildfires that occurred during the summers of 2016 and 2017. Estimates used to generate spatially explicit predictions of relative nest site selection are denoted as $\widehat \beta$, the median coefficient values of the posterior distribution. The term pd is the probability of direction statistic, used to indicate the proportion of the posterior distribution with the same size as $\widehat \beta$, while “Influence” represents the effect on habitat selection, where + indicates greater relative habitat selection closer to, or with increasing values of the feature, and - indicates lower relative habitat selection closer to, or with increasing higher values of the feature. Habitat predictors above the horizontal dividing line represent high certainty of effect, based on 95% credible intervals that did not overlap zero (2.5th and 97.5th percentiles had same sign).

^a^Exponential distance decay function; coefficient estimate represents proximity (nearness)

### Nest Survival

Due to a smaller effective sample size (e.g., 70 failed nests), habitat predictor covariates were subject to greater uncertainty in the estimation of their effects of nest survival. For example, the 95% CRIs for all predictor effects from the frailty model included 0 (Table 3). However, the following covariates had high probability (>0.85) for affecting nest survival, based on the probability of direction (*pd*; Makowski et al. 2019) or proportion of the posterior distribution having the same sign as the median β value: surface curvature (*r* = 75 m, *β* = −0.152, 95% CRI = −0.429–0.051, *pd* = 0.910), NDVI (*r* = 1451 m, *β* = −0.163, 95% CRI = −0.496–0.068, *pd* = 0.898), and sagebrush height (*r* = 439 m, *β* = −0.132, 95% CRI = −0.415–0.050, *pd* = 0.885). Each of these effects represented a negative influence on the hazard (i.e., reduced risk of nest failure) with increasing values of the covariate being more positively related to nest survival. In addition, nest survival was generally lower early in the season, based on the estimated effect of nest initiation date, and increased later in the season (Table 3). The study area predicted pre-fire 38-day cumulative nest survival surface is shown in Fig. 1. Posterior distributions of model coefficients are shown in Supplementary Materials S6 (Figs. S6–2).

**Table 3:** Posterior distribution estimates of habitat predictor covariates from a hierarchical model of nest survival (nest frailty) in the Virginia Mountains region of Nevada, USA

Habitat predictor	Scale	$\widehat \beta$ (50th)	2.5th	97.5th	*pd*	Influence	Source
Day of season		−0.264	−0.554	−0.018	0.985	+	Original data collection
Curvature	*r* = 75 m	−0.152	−0.429	0.051	0.910	+	https://viewer.nationalmap.gov/basic/
							https://evansmurphy.wixsite.com/evansspatial/arcgis-gradient-metrics-
Normalized difference vegetation index	*r* = 1451 m	−0.163	−0.496	0.068	0.898	+	10.5067/MODIS/MOD13Q1.0062015
Sagebrush height	*r* = 439 m	−0.132	−0.415	0.072	0.885	+	https://www.mrlc.gov/data
% Cover other sagebrush	*r* = 439 m	−0.080	−0.356	0.110	0.791	+	https://www.mrlc.gov/data
% Cover annual grass	*r* = 439 m	−0.073	−0.401	0.129	0.755	+	https://www.mrlc.gov/data
% Cover litter	*r* = 167 m	0.041	−0.146	0.285	0.684	−	https://www.mrlc.gov/data
% Burned area^b^	*r* = 167 m	0.039	−0.164	0.317	0.664	−	https://www.mtbs.gov
Topographic roughness	*r* = 1451 m	−0.020	−0.269	0.179	0.598	+	https://viewer.nationalmap.gov/basic/
							https://evansmurphy.wixsite.com/evansspatial/arcgis-gradient-metrics-
Medium-voltage power line	Exp. Decay^a^	−0.008	−0.475	0.409	0.528	+	https://www.spglobal.com/platts/en/products-services/electric-power
Hen age		−0.004	−0.397	0.375	0.512	+	Original data collection

Data were specific to the breeding seasons of study years 2009–2016, prior to significant wildfire events that occurred during the summers of 2016 and 2017. Estimates used to generate spatially explicit predictions of 38-day cumulative nest survival, converted from the hazard function of a nest frailty model, are denoted as $\widehat \beta$, the median coefficient values of the posterior distribution. The term pd is the probability of direction statistic, used to indicate the proportion of the posterior distribution with the same size as $\widehat \beta$, while “Influence” represents the effect on nest survival, where + indicates reduced hazard and greater survival closer to, or with increasing values of the feature, and - indicates increased hazard and lower survival closer to, or with increasing higher values of the feature.

^a^Exponential distance decay function; coefficient estimate represents proximity (nearness)

^b^Cumulative burned area represents effects of area burned during the previous 10 years

### Loss from Wildfire and Restoration of Nesting Habitat

**Fig. 2:**
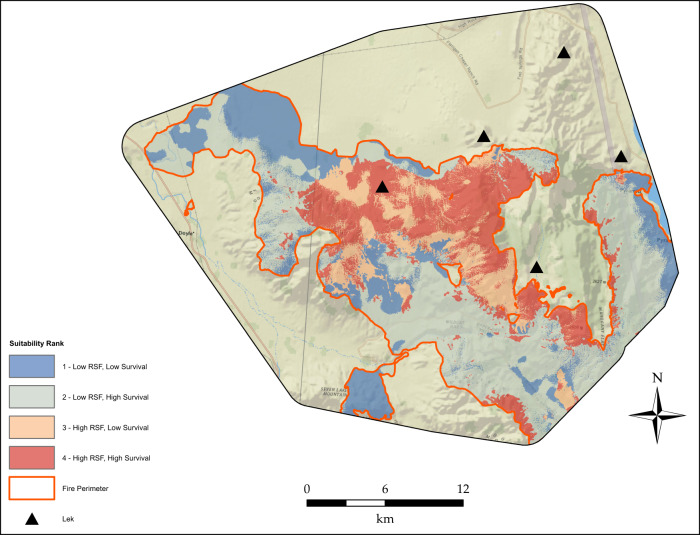
Habitat restoration index based on the intersection of loss of habitat selected by sage-grouse and loss of habitat contributions to nest survival following wildfire. Four classes were created by reclassifying the differenced nest Resource Selection Function (RSF) map based on relative losses in habitat selected pre-fire >0 (2 classes, low vs. high) and the differenced nest survival map based on the 50th percentile of loss of cumulative 38-day nest survival (2 classes, low vs. high)

Predicted losses of selected nesting habitat within the burned area approached nearly 100% of initial habitat values in some areas near existing leks (Fig. 1c), while the loss of potential nest survival probability within the burned area reached 0.6 and was consistently >0.1 throughout the burned area (Fig. 1d). The restoration index, representing four classes from low (1) to high (4) restoration suitability in terms of the habitat lost (i.e., restoration potential), revealed spatially explicit information to focus potential restoration efforts on areas where habitat quality was previously greatest prior to wildfire (Fig. 2).

**Fig. 3:**
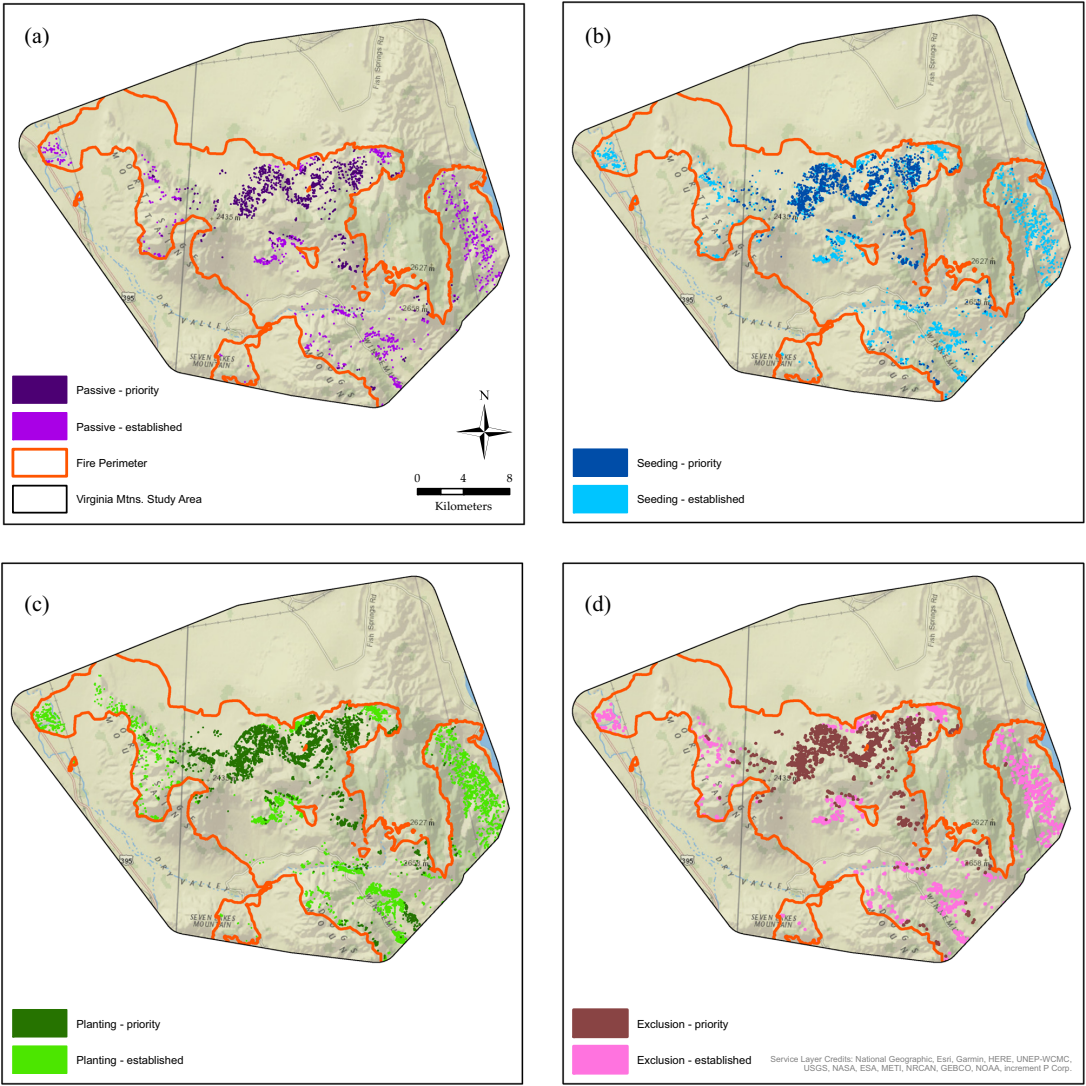
Established sagebrush recovery (>20% cover) within the 2016 and 2017 Virginia Mountain fires after 50 years under (**a**) passive, (**b**) seeding, (**c**) outplanting, and (**d**) grazing exclusion restoration efforts. Recovered sagebrush in priority nesting habitat is distinguished from non-priority recovery by darker shading

Following 30- and 50-years recovery within the wildfire perimeters, we identified 308 and 316 hectares of sagebrush that could be recovered through passive restoration, respectively, 399 and 410 hectares that could be recovered through grazing exclusion, respectively, 660 and 677 hectares that could be recovered through seeding, respectively, and 1367 and 2092 hectares that could be recovered through seedling transplanting within the entire burn scar after 30 and 50 years. Additionally, we combined the two highest classes of the restoration index with spatially explicit patterns of post-fire sagebrush recovery to identify areas with the highest potential for restoration success under different management scenarios (Fig. 3). With further refinement to focus on priority areas, passive restoration yielded 185 and 191 hectares, grazing exclusion yielded 249 and 257 hectares, seeding yielded 397 and 412 hectares, while planting seedlings yielded 832 and 1083 hectares after 30 and 50 years, respectively. Thus, active restoration approaches of seeding or planting seedlings produced nearly 2.1 and 4.5 times more habitat, respectively, than passively allowing recovery.

## Discussion

We provide a multi-scale decision-support framework that operationalizes concepts of ecological resilience to better inform implementation and effectiveness of passive and active restoration efforts through the lens of sage-grouse population performance (Chambers et al. 2017; Ricca et al. 2018; Ricca and Coates 2020). This quantitative framework helps minimize the spatial and temporal lag between traditional measures of ecosystem resilience and more immediate population responses to wildfire by combining fundamental soil and plant community processes with metrics of sage-grouse habitat selection and survival (Chambers et al. 2017; Ricca and Coates 2020). Our framework allows users to jointly prioritize areas that are crucial to sage-grouse populations and improve efficiency of restoration efforts by targeting areas with the highest capacity for recovery after wildfire. Our framework also provides the ability to estimate and compare the timeframe for expected outcomes of passive and active restoration projects to sage-grouse life history requirements and population trends. This model helps prioritize areas for the three restoration activities and given the high proportion of low resilience and resistance and the relatively slow growth rate of the sagebrush communities within this site, seeding and seedling transplanting will be required to expeditiously recover sage-grouse habitat across most of the site. Our recovery model can be refined as better estimates of seeding and transplanting recovery rates are established, as livestock grazing and exclusion impacts are quantified, and as improved measures of resilience and resistance are developed.

We accounted for four restoration options across a range of intervention efforts: passive, grazing exclusion, seeding, and seedling transplant. Excluding livestock following wildfire promotes the regrowth of native herbaceous vegetation that can compete with non-native invasive annual grasses (Reisner et al. 2013; Ellsworth et al. 2016; Condon and Pyke 2018), reduces additional disturbance to biocrust communities that infill the interspace that annual grasses colonize, and disrupts the spread of annual grass seed throughout the burn scar. Therefore, the primary benefit of our livestock exclusion scenario was an improved probability of sagebrush establishment, a benefit that would be realized within a few years post-wildfire and would not impose long-term grazing exclusion or significant costs to implement. Federal land management agencies often prevent livestock grazing on active allotments for two years following wildfire (U.S. Bureau of Land Management 2007; Williamson et al. 2020). However, additional exclusion time may be required as annual grass has been shown to continue to outcompete native herbaceous species up to twelve years post-wildfire (Miller et al. 2013).

The intention of sagebrush seeding is to replenish sagebrush seed availability and promote establishment across a broad extent (Pyke et al. 2020) and can be effective given cool, moist site conditions and seed mixes with species adapted to local conditions (Arkle et al.^ 2014^; Brabec et al. 2015; Richardson et al. 2015). However, seeding in warm and arid conditions can have low establishment success (Knutson et al. 2014; Shriver et al. 2019) and does not provide the more immediate benefits that transplanting sagebrush seedlings confers, such as increased cover and height advantages (Pyke et al. 2020). Based on our current recovery model, our results indicate transplanting is more useful for achieving accelerated habitat recovery to the required 20% sagebrush cover threshold within a time frame that is applicable to sage-grouse. These results generally align with empirical evidence presented in Pyke et al. (2020), who found that plots treated with seedling transplants met height requirements faster than seeded plots and that in order for seeding growth rates to match transplanted seedlings, survival must be equal. Our model can be refined as estimates of seeding and seedling transplant growth rates and resilience and resistance improve, with additional consideration given to their interactions. Our model also assumes seeding and seedling transplants are stocked from local sources and retain the adaptations to local climate and soil conditions of the pre-disturbance sagebrush community (Bower et al. 2014; Germino et al. 2019). Seed sources lacking local adaptations could limit establishment or survival (Brabec et al. 2015; Richardson et al. 2015; Germino et al. 2019).

While active restoration may be more efficient for restoring sage-grouse habitat, there are known cost and logistical constraints that can prohibit restoration over large extents (Dettweiler-Robinson et al. 2013; Grant-Hoffman and Plank 2021). Costs of seed treatments are impacted by seed density requirements, limited seed longevity, and seeding method (Shriver et al. 2018; Germino et al. 2018), while seedling transplant projects are restricted by seedling growing cost and availability (Grant-Hoffman and Plank 2021). From an economic perspective, high rate seeding may be an attractive alternative to transplanting to achieve sagebrush densification within restored patches (Pyke and Archer 1991). Pyke et al. 2020 found that seeding growth rates are competitive with transplanted seedlings under the assumption of equal survival. However, achieving nesting habitat restoration through seeding is difficult (Arkle et al. 2014). Due to low success (Knutson et al. 2014), the cost of seeding may ultimately meet or exceed the cost of transplanting (Boyd and Davies 2012; Dettweiler-Robinson et al. 2013; Pyke et al. 2020). The fine resolution of our selection and survival maps allow users to target and precisely delineate the areas that are predicted to have the greatest benefit for sage-grouse, potentially reducing the cost of treatment compared to delineating sites by other methods. Additionally, by downloading our prioritization map, users can refine priority areas with other GIS boundaries of interest such as land ownership, terrain profiles, distance to roads, etc. to customize a treatment site based on accessibility. Further, our simulations found that less costly management options such as grazing exclusion can effectively improve sagebrush recovery. Excluding grazing diminishes annual grass invasion which can increase sagebrush establishment by reducing competition through maintenance of soil-resource partitioning and reducing litter that can prevent sagebrush germination (Germino et al. 2016). For these reasons, the Bureau of Land Management currently employs grazing exclusion for 2 years following wildfire (U.S. Bureau of Land Management 2007). Grazing exclusion is not without cost as it will have immediate economic impacts on local ranching operations. However, strategically timed grazing may provide a management option to reduce economic losses. Fall-winter grazing has been shown to reduce non-native invasive annual grass cover and increase native perennial vegetation following fire (Davies et al. 2021).

Our finding that projected sage-grouse nest survival was negatively impacted by burned areas further highlights the need for active habitat restoration. Unfortunately, passive recovery of sagebrush to the minimum required cover for sage-grouse nests may lag behind habitat requirements to preserve sage-grouse populations (Arkle et al. 2014; Coates et al. 2016a; Anthony et al. 2021; Dudley et al. 2021), especially where increasing fire intensity severely degrades sagebrush canopy structure (Brooks et al. 2015). Compared to seedling transplants, seeding treatments can have lower establishment and slower growth (Arkle et al. 2014). Additionally, sage-grouse show strong fidelity to pre-fire nesting areas, despite apparent fitness consequences (Foster et al. 2018, O’Neil et al. 2020, Anthony et al. 2021). While recovery to pre-fire sagebrush conditions may take more than a decade even with restoration (Germino et al. 2018; Pyke et al. 2020), active efforts to help accelerate recovery could serve to minimally meet life history requirements to sustain local populations. Our approach considers sagebrush to be successfully restored at the minimal cover required for sage-grouse (Connelly et al. 2000; Smith et al. 2020). Application of our restoration suitability framework can reduce the temporal lag between resilience and habitat quality according to this benchmark. Spatially explicit frameworks like ours help to identify sites where restoration would be most effective for nest selection and survival and can guide the placement of islands of habitat (Hulvey et al. 2017). We modeled transplant recovery rates based on current densities of 1 plant m^−2^ (Wirth and Pyke 2011). However, the degree to which pre-fire sagebrush cover is restored will further depend on the density of transplants (Pyke et al. 2020) and increasing the density of transplants can decrease recovery time (Pyke et al. 2020). Our quantitative framework can be adjusted to reflect planned densities as recovery rates for different seedling densities are established.

The framework we have outlined implements some of the proposed improvements discussed in Ricca and Coates (2020) to other models of operationalized resilience that incorporate resilience and resistance into conservation planning for sage-grouse habitat and distribution restoration (e.g., Chambers et al. 2017; Barnard et al. 2019). We explicitly modeled the post-restoration impact on both sage-grouse nest selection and survival by predicting sagebrush community specific recovery under scenarios of both passive and active restoration. In addition, our framework mitigates potential for incomplete predictions of habitat by combining predicted surfaces of nest selection and nest survival into a single index of restoration suitability that captures potential for species reproduction in addition to distribution. This allows users to rank potential restoration sites not only by selection/occupancy, but also by metrics such as survival that are often more representative of true habitat quality (Gaillard et al. 2010). In this way, the framework facilitates consideration of source-sink dynamics (Matthiopoulos et al. 2015) and the potential for ecological traps, which contain detrimental habitat components alongside attractive components which are selected by sage-grouse (Aldridge and Boyce 2007; Coates et al. 2017; Foster et al. 2018; Heinrichs et al. 2018; O’Neil et al. 2020). Notably, we prioritized restoration categories based on selection patterns, above survival patterns, because these areas are most likely to be used, and thus restoration would have the greatest potential to improve habitat for the largest number of birds as well as potentially restore areas that may have previously served as traps. This framework can also be used with spatially explicit layers that quantify overall ecosystem health rather than a sensitive species of interest, such as ecosystem resilience and resistance, soil characteristics, or disturbance response information (Ricca and Coates 2020).

### Caveats for Use

This study was not without constraints. Although this framework focuses on nest habitat, including responses from other sage-grouse life history stages will likely provide a more complete picture of predicted restoration effectiveness on population performance. For example, recent evidence suggested that wildfire was responsible for reduced adult female survival in Oregon (Foster et al. 2018). Our framework can be adapted to incorporate survival and other modeled parameters across multiple life stages contingent on data availability. The methodological framework can be modified for other sagebrush-obligate species that might not fall under the umbrella of sage-grouse conservation (Hanser and Knick 2011; Carlisle et al. 2018) conditional upon the availability of spatially explicit data (Suding 2011), where occupancy and/or fitness information is available. Lastly, because recent evidence suggests that sage-grouse functional responses to vegetation components vary among sagebrush ecosystems at different spatial and temporal extents, such as Great Basin (Coates et al. 2020; Smith et al. 2020) and range-wide (Doherty et al. 2016), our modeled parameters may be unique to our study area. However, the increasing availability of spatially explicit sage-grouse vital rate data and access to multi-scale landscape covariates promotes expansion of our framework into a generalizable model that incorporates multiple sites and years (O’Neil et al. 2020, Ricca and Coates 2020). Such a generalizable model would allow users to evaluate where and how to carry out restoration strategies immediately following wildfire in areas without pre-wildfire data.

Our framework specifically focuses on disturbance from wildfire and does not explicitly account for additional disturbances such as grazing or soil surface disruption. There is evidence of increased grazing pressure following fire, even with rest from grazing (Condon and Pyke 2018), and understanding the historic grazing regime of our site could better indicate the potential for transitions to alternative states. Due to data limitations on grazing regimes across our site, we were unable to assess pre-fire grazing impacts on nest selection and survival, or the potential for post-fire grazing to delay the recovery of perennial grasses that provide nesting habitat for sage-grouse or increase non-native invasive species cover (Fisher et al. 2009; Souther et al. 2019). Our post-fire sage-grouse selection and survival models assumed exclusion from grazing across the study site, simulating partial recovery of perennial grasses nearly immediately (<5 years) after the wildfires occurred (Arkle et al. 2014). The framework could be greatly improved with the availability of data that quantify direct and indirect impacts of livestock grazing specific to perennial grass and sagebrush recovery. This is dependent on spatially explicit records of grazing timing, intensity, and history on the landscape. Also needed are data that quantify the response of plant communities (i.e., percent cover and height increases) to grazing exclusion, especially in areas with concomitant disturbances such as wildfire. Additionally, our model prioritized sagebrush recovery for nesting habitat and did not estimate the value of the sagebrush community composition on additional life history requirements such as winter forage (Frye et al. 2013; Fremgen-Tarantino et al. 2020), although our threshold of 20% cover also pertains to winter habitat needs (Connelly et al. 2000; Fremgen-Tarantino et al. 2020). Additionally, a growing body of literature indicates that biocrusts are a major component in passive recovery of sagebrush steppe (Su et al. 2009; Condon and Pyke 2020). Because the models of surface cover (Xian et al. 2015) available in our study area did not estimate biocrust, we relied on resistance and resilience classes to quantify the ecological capacity which are derived from similar soil components and are currently a standard measure of operational resilience (Chambers et al. 2019, Ricca and Coates 2020). Remote sensing models to detect surface cover of biocrust need to be developed to provide a more nuanced estimate of recovery in future decision-support tools.

Our metrics of ecological resilience could be supplemented with data derived from more rigorous monitoring of restoration outcomes. The resilience and resistance mapping used in our study (Maestas et al. 2016) reflected static profiles, but recent evidence suggest that these are projected to shift at an approximated date of 2070 towards warmer and wetter soil types (higher soil temperature, more cool season moisture) in Great Basin sagebrush (Bradford et al. 2019). Such a shift will result in more moderate resilience and resistance and less high and low resilience and resistance. Incorporating decadal outcomes derived from more realistic maps that depict temporal shifts would be valuable as these spatial layers become available.

In conclusion, we have developed a quantitative decision-support framework that allows users to evaluate predicted effectiveness of restoration strategies aimed at reducing immediate impacts of wildfire to sage-grouse populations. Our framework operationalizes concepts of resilience and resistance to facilitate rapid return of sagebrush in areas that are most conducive to nesting, which might otherwise result in ecological traps based on sage-grouse nest site fidelity. This framework has broader decision-support applications within the sagebrush ecosystem. Use of this quantitative framework can be expanded to other sagebrush-obligate species with the development of predictive surfaces of their population dynamics. While the applications presented herein were specific to wildfire, this framework is readily adaptable to various drivers of habitat loss within sagebrush ecosystems as it quantifies operational resilience irrespective of the type of catastrophic disturbance that has occurred. Therefore, users can adapt this framework to guide land reclamation or mitigation actions in response to other disturbances to the sagebrush ecosystem such as agricultural expansion, energy development, and unsustainable livestock grazing practices.

## Supplementary information

Supplementary Information

## Data Availability

Data supporting the results of this analysis will be madeavailable to the public via the USGS ScienceBase data repository (data; sciencebase.gov; 10.5066/P96K6X05; Roth et al. 2022).

## Code Availability

Code is made available as supplemental material with this manuscript.

## References

[CR1] Abatzoglou JT, Park WA (2016). Impact of anthropogenic climate change on wildfire across western US forests. Proc Natl Acad Sci USA.

[CR2] Aldridge CL, Boyce MS (2007). Linking occurrence and fitness to persistence: habitat-based approach for endangered Greater Sage-Grouse. Ecol Appl.

[CR4] Anthony, CR, Foster, LJ, Hagen, CA, Dugger, KM (2021) Acute and lagged fitness consequences for a sagebrush obligate in a post mega-wildfire landscape. Ecol Evol 10.1002/ece3.8488 10.1002/ece3.8488 PMC8794719 35127022

[CR5] Arkle RS, Pilliod DS, Hanser SE, Brooks ML, Chambers JC, Grace JB, Knutson KC, Pyke DA, Welty JL, Wirth TA (2014). Quantifying restoration effectiveness using multi-scale habitat models: implications for sage-grouse in the Great Basin. Ecosphere.

[CR6] Baker WL (2006). Fire and restoration of sagebrush ecosystems. Wildl Soc Bull.

[CR7] Baker WL, Knick ST, Connelly JW (2011). Pre‐Euro‐American and recent fire in sagebrush ecosystems. Greater sage‐grouse: ecology and conservation of a landscape species and its habitats. Studies in Avian Biology. Vol 38.

[CR8] Barnard DM, Germino MJ, Arkle RS, Bradford JB, Duniway MC, Pilliod DS, Pyke DA, Shriver RK, Welty JL (2019). Soil characteristics are associated with gradients of big sagebrush canopy structure after disturbance. Ecosphere.

[CR9] Bates Jonathan D, Boyd Chad S, Davies Kirk W (2020). Longer-term post-fire succession on Wyoming big sagebrush steppe. Int J Wildland Fire.

[CR10] Beck JL, Connelly J, Reese K (2009). Recovery of Greater Sage-Grouse habitat features in Wyoming big sagebrush following prescribed fire. Restor Ecol.

[CR11] Belnap J, Reynolds RL, Reheis MC, Phillips SL, Urban FE, Goldstein HL (2009). Sediment losses and gains across a gradient of livestock grazing and plant invasion in a cool, semi-arid grassland, Colorado Plateau, USA. Aeolian. Research.

[CR12] Bower AD, Clair J, Erickson V (2014). Generalized provisional seed zones for native plants. Ecol Appl.

[CR13] Boyd CS, Davies KW (2012). Spatial variability in cost and success of revegetation in the Wyoming big sagebrush community. Environ Manag.

[CR14] Boyte SP, Wylie BK (2018) Near-real-time herbaceous annual cover in the sagebrush ecosystem, USA, July 2018. U.S. Geological Survey data release. 10.5066/P96PVZIF

[CR15] Brabec MM, Germino MJ, Shinneman DJ, Pilliod DS, McIlroy SK, Arkle RS (2015). Challenges of establishing big sagebrush (Atemisia tridentata) in rangeland restoration: effects of herbicide, mowing, whole-community seeding, and sagebrush seed sources. Rangel Ecol Manag.

[CR16] Bradford JB, Schlaepfer DR, Lauenroth WK, Palmquist KA, Chambers JC, Maestas JD, Campbell SB (2019). Climate-driven shifts in soil temperature and moisture regimes suggest opportunities to enhance assessments of dryland resilience and resistance. Front Ecol Evolution.

[CR17] Bradley BA, Curtis CA, Fusco EJ, Abatzoglou JT, Balch JK, Dadashi S, Tuanmu MN (2018). Cheatgrass (Bromus tectorum) distribution in the intermountain Western United States and its relationship to fire frequency, seasonality, and ignitions. Biol invasions.

[CR18] Brooks ML, Matchett JR, Shinneman DJ, Coates PS (2015) Fire patterns in the range of the greater sage-grouse, 1984-2013—Implications for conservation and management. US Geological Survey

[CR19] Bureau of Land Management (2020) Greater sage-grouse resource management plan revisions and amendments. https://eplanning.blm.gov/eplanning-ui/project/90121

[CR20] Carlisle JD, Keinath DA, Albeke SE, Chalfoun AD (2018). Identifying holes in the greater sage‐grouse conservation umbrella. J Wildl Manag.

[CR21] Chambers JC, Brooks ML, Germino MJ, Maestas JD, Board DI, Jones MO, Allred BW (2019). Operationalizing resilience and resistance concepts to address invasive grass-fire cycles. Front Ecol Evolution.

[CR22] Chambers JC, Bradley BA, Brown CS, D’Antonio C, Germino MJ, Grace JB, Hardegree SP, Miller RF, Pyke DA (2014). Resilience to stress and disturbance, and resistance to Bromus tectorum L. invasion in cold desert shrublands of western North America. Ecosystems.

[CR23] Chambers JC, Beck JL, Bradford JB, Bybee J, Campbell S, Carlson J, Christiansen TJ, Clause KJ, Collins G, Crist MR, Dinkins JB, Doherty KE, Edwards F, Espinosa S, Griffin KA, Griffin P, Haas JR, Hanser SE, Havlina DW, Henke KF, Hennig JD, Joyce LA, Kilkenny FM, Kulpa SM, Kurth LL, Maestas JD, Manning M, Mayer KE, Mealor BA, McCarthy C, Pellant M, Perea MA, Prentice KL, Pyke DA, Wiechman LA, Wuenschel A (2017) Science framework for conservation and restoration of the sagebrush biome: linking the Department of the Interior’s Integrated Rangeland Fire Management Strategy to long-term strategic conservation actions. Gen. Tech. Rep. RMRS-GTR-360. Fort Collins, CO: US Department of Agriculture, Forest Service, Rocky Mountain Research Station. 213 p. 360

[CR24] Coates PS, Prochazka BG, Ricca MA, Gustafson KB, Ziegler P, Casazza ML (2017). Pinyon and juniper encroachment into sagebrush ecosystems impacts distribution and survival of greater sage-grouse. Rangel Ecol Manag.

[CR25] Coates PS, Casazza ML, Blomberg EJ, Gardner SC, Espinosa SP, Yee JL, Weichman L, Halstead BJ (2013). Evaluating greater sage‐grouse seasonal space use relative to leks: Implications for surface use designations in sagebrush ecosystems. J Wildl Manag.

[CR26] Coates PS, Ricca MA, Prochazka BG, Brooks ML, Doherty KE, Kroger T, Blomberg EJ, Hagen CA, Casazza ML (2016). Wildfire, climate, and invasive grass interactions negatively impact an indicator species by reshaping sagebrush ecosystems. Proc Natl Acad Sci USA.

[CR27] Coates PS, Brussee BE, Ricca MA, Severson JP, Casazza ML, Gustafson KB, Espinosa SP, Gardner SC, Delehanty DJ (2020). Spatially-explicit models of seasonal habitat for greater sage-grouse at broad spatial scales: informing areas for management in Nevada and northeaster California. Ecol Evolution.

[CR28] Coates PS, Prochazka BG, O’Donnell MS, Aldridge CL, Edmunds DR, Monroe AP, Ricca MA, Wann GT, Hanser SE, Wiechman LA, Chenaille M (2021). Range-wide greater sage-grouse hierarchical monitoring framework—Implications for defining population boundaries, trend estimation, and a targeted annual warning system.

[CR29] Coates PS, Casazza ML, Ricca MA, Brussee BE, Blomberg EJ, Gustafson KB, Overton CT, Davis DM, Niell LE, Espinosa SP, Gardner SC, Delehanty DJ (2016). Integrating spatially explicit indices of abundance and habitat quality: an applied example for greater sage-grouse management. J Appl Ecol.

[CR30] Condon LA, Pyke DA (2018). Fire and grazing influence site resistance to *Bromus tectorum* through their effects on shrub, bunchgrass and biocrust communities in the Great Basin (USA). Ecosystems.

[CR31] Condon LA, Pyke DA (2020). Components and predictors of biological soil crusts vary at the regional vs. plant community scales. Front Ecol Evolution.

[CR32] Connelly JW, Schroeder MA, Sands AR, Braun CE (2000). Guidelines to manage sage grouse populations and their habitats. Wildl Soc Bull.

[CR33] Connelly JW, Knick ST, Braun CE, Baker WL, Beever EA, Christiansen T, Doherty KE, Garton EO, Hanser SE, Johnson DH, Leu M, Miller, RF, Naugle DE, Oyler-McCance SJ, Pyke DA, Walker KBL, Wisdom MJ (2011) Conservation of greater sage-grouse: a synthesis of current trends and future management. In: Knick ST, Connelly JW (eds). Greater sage-grouse: ecology and conservation of a landscape species and its habitats. Studies in Avian Biology No. 38, University of California Press, pp 549–563

[CR34] Connelly JW, Knick ST, Schroeder MA, Stiver SJ, Western association of fish and wildlife agencies (2004) Conservation assessment of greater sage-grouse and sagebrush habitats. All US Government Documents (Utah Regional Depository) 73

[CR35] Converse SJ, Royle JA, Adler PH, Urbanek RP, Barzen JA (2013). A hierarchical nest survival model integrating incomplete temporally varying covariates. Ecol Evolution.

[CR36] Cooper SV, Lesica, Kudray GM (2011). Post-fire recovery of Wyoming big sagebrush steppe in central and southeast Montana. Nat Resour Environ Issues.

[CR37] Copeland SM, Munson SM, Pilliod DS, Welty JL, Bradford JB, Butterfield BJ (2018). Long-term trends in restoration and associated land treatments in the southwestern United States: Vegetation treatment trends related to restoration. Restor Ecol.

[CR38] Crist MR, Chambers JC, Philips SL, Prentice KL, Wiechman LA. (2019) Science framework for conservation and restoration of the sagebrush biome: Linking the Department of the Interior’s Integrated Rangeland Fire Management Strategy to long-term strategic conservation actions. Part 2. Management applications. Management Applications. Gen. Tech. Rep. RMRS-GTR-389. Fort Collins, CO: US Department of Agriculture, Forest Service, Rocky Mountain Research Station 93303

[CR39] D’Antonio CM, Vitousek PM (1992). Biological invasions by exotic grasses, the grass/fire cycle, and global change. Annu Rev Ecol Syst.

[CR40] Davies KW, Bates JD, Perryman B, Arispe S (2021). Fall-winter grazing after fire in annual grass invaded sagebrush steppe reduced annuals and increased native bunchgrass. Rangel Ecol Manag.

[CR41] Dettweiler-Robinson E, Bakker JD, Evans JR, Newsome H, Davies GM, Wirth TA, Pyke DA, Easterly RT, Salstrom D, Dunwiddle PW (2013). Outplanting wyoming big sagebrush following wildfire: stock performance and economics. Rangel Ecol Manag.

[CR42] Doherty KE, Evan JS, Coates PS, Juliusson LM, Fedy BC (2016). Importance of regional variation in conservation planning: a rangewide example of the Greater Sage-Grouse. Ecosphere.

[CR43] Dudley, IF, Coates, PS, Prochazka, BG, O’Neil, ST, Gardner, S, Delehanty, DJ (2021) Large-scale wildfire reduces population growth in a peripheral population of sage-grouse. Fire Ecology 17(15) 10.1186/s42408-021-00099-z

[CR44] Eldridge DJ, Poore AGB, Ruiz-Colmenero M, Letnic M, Soliveres S (2016). Ecosystem structure, function, and composition in rangelands are negatively affected by livestock grazing. Ecol Appl.

[CR45] Ellsworth LM, Wrobleski DW, Kauffman JB, Reis SA (2016). Ecosystem resilience is evident 17 years after fire in Wyoming big sagebrush ecosystems. Ecosphere.

[CR46] Fieberg J, Signer J, Smith B, Avgar TA (2021). A ‘How to’ guide for interpreting parameters in habitat selection analyses. J Anim Ecol.

[CR47] Fisher J, Cole KL, Anderson RS (2009). Using packrat middens to assess grazing effects on vegetation change. J Arid Environ.

[CR48] Flannigan MD, Krawchuk MA, Groot WJ, Wotton BM, Gowman LM (2009) Implications of changing climate for global wildland fire. International Journal of Wildland Fire 18(5):483 10.1071/WF08187

[CR49] Foster LJ, Dugger KM, Hagen CA, Budeau DA (2018). Greater sage‐grouse vital rates after wildfire. J Wildl Manag.

[CR50] Fremgen-Tarantino MR, Peña JJ, Connelly JW, Sorensen Forbey J (2020) Winter foraging ecology of Greater Sage-Grouse in a post-fire landscape. J Arid Environ 178

[CR51] Frye GG, Connelly JW, Musil DD, Forbey JS (2013). Phytochemistry predicts habitat selection by an avian herbivore at multiple spatial scales. Ecology.

[CR52] Gaillard JM, Hebblewhite M, Loison A, Fuller M, Powell R, Basille M, Van Moorter B (2010). Habitat–performance relationships: finding the right metric at a given spatial scale. Philos Trans R Soc B: Biol Sci.

[CR53] Garton EO, Connelly JW, Hagen CA, Horne JS, Moser A, Schroeder MA (2011) Greater sage-grouse population dynamics and probability of persistence. In: Knick ST, Connelly JW (eds). Greater sage-grouse: ecology and conservation of a landscape species and its habitats. Studies in Avian Biology No. 38, University of California Press, pp. 293–382

[CR54] Germino MJ, Moser AM, Sands AR (2019). Adaptive variation, including local adaptation, requires decades to become evident in common gardens. Ecol Application.

[CR55] Germino MJ, Barnard DM, Davidson BE, Arkle RS, Pilliod DS, Fisk MR, Applestein C (2018) Thresholds and hotspots for shrub restoration following a heterogeneous megafire. Landscape Ecology 33:1177–1194

[CR56] Germino MJ, Belnap J, Stark JM, Allen EB, Rau B (2016) Ecosystem impacts of exotic annual invaders in the genus Bromus. In: Exotic brome-grasses in arid and semiarid ecosystems of the Western US. Springer pp. 61–95.

[CR57] Giesen KM, Schoenberg TJ, Braun CE (1982) Methods for trapping sage grouse in Colorado. Wildlife Society Bulletin 224–231

[CR58] Gillies CS, Hebblewhite M, Nielson SE, Krawchuk MA, Aldridge CL, Frair JL, Saher DJ, Stevens CE, Jerde CL (2006). Application of random effects to the study of resource selection by animals. J Anim Ecol.

[CR59] Grant-Hoffman MN, Plank HL (2021). Practical postfire sagebrush shrub restoration techniques. Rangel Ecol Manag.

[CR60] Halstead BJ, Wylie GD, Coates PS, Valcarcel P, Casazza ML (2012). Bayesian shared frailty models for regional inference about wildlife survival. Anim Conserv.

[CR61] Hanser SE, Knick ST (2011) Greater sage-grouse as an umbrella species for shrubland passerine birds: a multiscale assessment. In: Knick ST, Connelly JW (eds). Greater sage-grouse: ecology and conservation of a landscape species and its habitats. Studies in Avian Biology No. 38, University of California Press, pp 475– 488

[CR62] Heinrichs JA, Aldridge CL, Gummer DL, Monroe AP, Schumaker NH (2018). Prioritizing actions for the recovery of endangered species: Emergent insights from Greater Sage-grouse simulation modeling. Biol Conserv.

[CR63] Holloran MJ, Anderson SH (2005). Spatial distribution of greater sage-grouses in relatively contiguous sagebrush habitats. Condor.

[CR64] Hulvey KB, Leger EA, Porensky LM, Roche LM, Veblen KE, Fund A, Shaw J, Gornish ES (2017). Restoration islands: a tool for efficiently restoring dryland ecosystems?. Restor Ecol.

[CR65] Johnson CJ, Nielsen SE, Merrill EH, McDonald TL, Boyce MS (2006). Resource selection functions based on use–availability data: theoretical motivation and evaluation methods. J Wildl Manag.

[CR66] Kellner K (2018) jagsUI: a wrapper around ‘rjags’ to streamline ‘JAGS’ analyses

[CR67] Knick ST, Connelly JW (eds) (2011). Greater sage-grouse: ecology and conservation of a landscape species and its habitats. Studies in Avian Biology No. 38, University of California Press

[CR68] Knutson KC, Pyke DA, Wirth TA, Arkle RS, Pilliod DS, Brooks ML, Chambers JC, Grace JB (2014). Long‐term effects of seeding after wildfire on vegetation in Great Basin shrubland ecosystems. J Appl Ecol.

[CR69] Kolada EJ, Sedinger JS, Casazza ML (2009). Nest site selection by greater sage-grouse in Mono County, California. J Wildl Manag.

[CR70] Lesica P, Cooper SV, Kudray G (2007). Recovery of big sagebrush following fire in southwest Montana. Rangel Ecol Manag.

[CR71] Lockyer ZB, Coates PS, Casazza ML, Espinosa S, Delehanty DJ (2015) Nest‐site selection and reproductive success of greater sage‐grouse in a fire‐affected habitat of northwestern Nevada. The Journal of Wildlife Management 79:785–797

[CR72] Maestas JD, Campbell SB, Chambers JC, Pellant M, Miller RF (2016). Tapping soil survey information for rapid assessment of sagebrush ecosystem resilience and resistance. Rangelands.

[CR73] Matthiopoulos J, Fieberg J, Aarts G, Beyer HL, Morales JM, Haydon DT (2015) Establishing the link between habitat selection and animal population dynamics. Ecological Monographs, 85:413–436. 10.1890/14-2244.1

[CR74] McAdoo JK, Boyd CS, Sheley RL (2013). Site, competition, and plant stock influence transplant success of Wyoming big sagebrush. Rangel Ecol Manag.

[CR75] Meinke CW, Knick ST, Pyke DA (2009). A spatial model to prioritize sagebrush landscapes in the Intermountain West (USA) for restoration. Restor Ecol.

[CR76] Miller JR, Hobbs RJ (2007). Habitat restoration—Do we know what we’re doing?. Restor Ecol.

[CR77] Miller RF, Chambers JC, Pyke DA, Pierson FB, Williams CJ (2013). A review of fire effects on vegetation and soils in the Great Basin Region: response and ecological site characteristics. Gen. Tech. Rep. RMRS-GTR-308.

[CR78] Moffet CA, Taylor JB, Booth DT (2015). Postfire shrub cover dynamics: A 70-year fire chronosequence in mountain big sagebrush communities. J Arid Environ.

[CR79] Nelle PJ, Reese KP, Connelly JW (2000). Long-term effects of fire on sage grouse habitat. J Rangel Manag.

[CR80] Nelson ZJ, Weisberg PJ, Kitchen SG (2014). Influence of climate and environment on post‐fire recovery of mountain big sagebrush. Int J Wildland Fire.

[CR81] Northrup JM, Hooten MN, Anderson CR, Wittemyer G (2013). Practical guidance on characterizing availability in resource selection functions under a use-availability design. Ecology.

[CR82] O’Connor RC, Germino MJ, Barnard DM, Andrews CM, Bradford JB, Pilliod DS, Arkle RS, Shriver RK (2020). Small-scale water deficits after wildfires create long-lasting ecological impacts. Environ Res Lett.

[CR83] O’Neil ST, Coates PS, Brussee BE, Ricca MA, Espinosa SP, Garder SC, Delehanty DJ (2020) Wildfire and the ecological niche: diminishing habitat suitability for an indicator species within semi-arid ecosystems. Global Change Biology 10.1111/gcb.15300 PMC7693117 32741106

[CR84] Park T, Casella G (2008) The Bayesian lasso. Journal of the American Statistical Association 103:681–686 10.1198/016214508000000337

[CR85] Perring MP, Standish RJ, Price JN, Craig MD, Erickson TE, Ruthrof KX, Whiteley AS, Valentine LE, Hobbs RJ (2015). Advances in restoration ecology: rising to the challenges of the coming decades. Ecosphere.

[CR86] Pilliod DS, Welty JL, Arkle RS (2017). Refining the cheatgrass–fire cycle in the Great Basin: Precipitation timing and fine fuel composition predict wildfire trends. Ecol evolution.

[CR87] Plummer M (2003) JAGS: A program for analysis of Bayesian graphical models using Gibbs sampling. In: Proceedings of the 3rd international workshop on distributed statistical computing.Vol. 124 Vienna, Austria p. 125

[CR88] Plummer M (2018) rjags: Bayesian graphical models using MCMC.

[CR89] Pyke DA, Chambers JC, Pellant M, Knick ST, Miller RF, Beck JL, Doescher PS, Schupp EW, Roundy BA, Brunson M, McIver JD (2015). Restoration handbook for sagebrush steppe ecosystems with emphasis on greater sage-grouse habitat-Part 1. Circular 1416.

[CR90] Pyke DA, Archer S (1991) Plant–plant interactions affecting plant establishment and persistence on revegetated rangeland. Journal of Range Management 44:550–557

[CR91] Pyke DA, Shriver RK, Arkle RS, Pilliod DS, Aldridge CL, Coates PS, Germino MJ, Heinrichs JA, Ricca MA, Shaff SE (2020) Postfire growth of seeded and planted big sagebrush—strategic designs for restoring greater sage-grouse nesting habitat. Restor Ecol 28: 1495–1504 10.1111/rec.13264

[CR92] Reisner MD, Grace JB, Pyke DA, Doescher PS (2013). Conditions favoring *Bromus tectorum* dominance of endangered sagebrush steppe ecosystems. J Appl Ecol.

[CR93] Ricca MA, Coates PS (2020). Integrating ecosystem resilience and resistance into decision support tools for multi-scale population management of a sagebrush indicator species. Front Ecol Evolution.

[CR94] Ricca MA, Coates PS, Gustafson KB, Brussee BE, Chambers JC, Espinosa SP, Gardner SC, Lisius S, Zielgler P, Delehandty DJ, Casazza ML (2018). A conservation planning tool for Greater Sage‐grouse using indices of species distribution, resilience, and resistance. Ecol Appl.

[CR95] Richardson BA, Ortiz HG, Carlson SL, Jaeger DM, Shaw NL (2015). Genetic and environmental effects on seed weight in subspecies of big sagebrush: applications for restoration. Ecosphere.

[CR96] Rollins MG (2009). LANDFIRE: a nationally consistent vegetation, wildland fire, and fuel assessment. Int J Wildland Fire.

[CR97] Roth CL, O’Neil ST, Coates PS, Ricca MA, Pyke DA, Aldridge CL, Heinrichs JA, Espinosa SP, Delehanty DJ. (2022) An index for prioritizing post-wildfire restoration in sage-grouse habitat, U.S. Geological Survey, 10.5066/P96K6X05

[CR98] Row JR, Fedy BC (2017) Spatial and temporal variation in the range-wide cyclic dynamics of greater sage-grouse. Oecologia 185:687–698 10.1007/s00442-017-3970-9 29052009

[CR99] Rowland MM, Wisdom MJ, Suring L (2006). Greater sage-grouse as an umbrella species for sagebrush-associated vertebrates. Biol Conserv.

[CR100] Shinneman DJ, McIlroy SK (2016). Identifying key climate and environmental factors affecting rates of post-fire big sagebrush (Artemisia tridentata) recovery in the northern Columbia Basin, USA. Int J Wildland Fire.

[CR101] Shinneman DJ (2020) North American Sagebrush Steppe and Shrubland: Reference Module in Earth Systems and Environmental Sciences, 10.1016/B978-0-12-409548-9.11982-7

[CR102] Shriver RK, Andrews CM, Pilliod DS, Arkle RS, Welty JL, Germino MJ, Duniway MC, Pyke DA, Bradford JB (2018). Adapting management to a changing world: Warm temperatures, dry soil, and interannual variability limit restoration success of a dominant woody shrub in temperate drylands. Glob Change Biol.

[CR103] Shriver RK, Andrews CM, Arkle RS, Barnard DM, Duniway MC, Germino MJ, Pilliod DS, Pyke DA, Welty JL, Bradford JB (2019). Transient population dynamics impede restoration and may promote ecosystem transformation after disturbance. Ecol Lett.

[CR104] Smith JT, Allred BW, Boyd CS, Carlson JC, Davies KW, Hagen CA, Naugle DE, Olsen AC, Tack JD (2020). Are sage‐grouse fine‐scale specialists or shrub‐steppe generalists?. J Wildl Manag.

[CR105] Souther S, Loeser M, Crews TE, Sisk T (2019) Complex response of vegetation to grazing suggests need for coordinated, landscape-level approaches to grazing management. Global Ecology and Conservation 20

[CR106] Su YG, Li XR, Zheng JG, Huang G (2009). The effect of biological soil crusts of different successional stages and conditions on the germination of seeds of three desert plants. J Arid Environ.

[CR107] Suding KN (2011) Toward an era of restoration in ecology: successes, failures, and opportunities ahead. Annual review of ecology, evolution, and systematics 42

[CR108] Taylor RL, Walker BL, Naugle DE, Mills SL (2012). Managing multiple vital rates to maximize greater sage-grouse population growth. J Wildl Manag.

[CR109] U.S. Department of the Interior (2017) An Integrated Rangeland Fire Management Strategy: Final Report to the Secretary of the Interior. Washington, D.C.: Rangeland Fire Task Force.

[CR110] U.S. Department of the Interior Bureau of Land Management (2007) Burned area emergency stabilization and rehabilitation handbook. BLM Handbook H-1742

[CR119] U.S. Fish and Wildlife Service (2015) Endangered and threatened wildlife and plants; 12-month finding on a petition to list the greater sage-grouse (centrocercus urophasianus) as an endangered or threatened species. Proposed Rule Fed Register 80:59858–59942

[CR111] Wakkinen WL, Resse KP, Connelly JW, Fischer RA (1992) An improved spotlighting technique for capturing sage-grouse. Wildife Society Bulletin 20

[CR112] Wambolt CL, Walhof KS, Frisina MR (2001). Recovery of big sagebrush communities after burning in south‐western Montana. J Environ Manag.

[CR113] Watts MJ, Wambolt CL (1996). Long‐term recovery of Wyoming big sagebrush after four treatments. J Environ Manag.

[CR114] Wijayratne UC, Pyke DA (2012). Burial increases seed longevity of two Artemisia tridentata (Asteraceae) subspecies. Am J Bot.

[CR115] Williamson MA, Fleishman E, MacNally RC, Chambers JC, Bradley BA, Dobkin DS, Board DI, Fogarty FA, Horning N, Leu M, Zillig MW (2020). Fire, livestock grazing, precipitation, and topography affect occurrence and prevalence of cheatgrass (*Bromus tectorum*) in the central Great Basin, USA. Biol Invasions.

[CR116] Wirth TA, Pyke DA (2011). Effectiveness of post-fire seeding at the Fitzner-Eberhardt Arid Land Ecology Reserve.

[CR117] Xian G, Homer C, Rigge M, Shi H, Meyer D (2015). Characterization of shrubland ecosystem components as continuous fields in the northwest United States. Remote Sens Environ.

[CR118] Ziegenhagan LL, Miller RF (2009). Postfire recovery of two shrubs in the interiors of large burns in the Intermountain West, USA. West North Am Naturalist.

