# Supplementary material for: Targeting Sagebrush (Artemisia Spp.) Restoration Following Wildfire with Greater Sage-Grouse (Centrocercus Urophasianus) Nest Selection and Survival Models
Source: Environ Manage. 2022 Jun 10;70(2):288–306. doi: 10.1007/s00267-022-01649-0 (PMC9252971; doi:10.1007/s00267-022-01649-0)
Supplement: Supplementary file 1 — Supplementary Information [file 267_2022_1649_MOESM1_ESM.docx]

**Title:** Targeting sagebrush (*Artemisia* spp.) restoration following wildfire with Greater Sage-Grouse (*Centrocercus urophasianus*) nest selection and survival models

**Journal:** Environmental Management

**Authors:**Cali L. Roth, Shawn T. O’Neil, Peter S. Coates^1^, Mark A. Ricca, David A. Pyke, Cameron L. Aldridge, Julie A. Heinrichs, Shawn P. Espinosa, David J. Delehanty

^1^U.S. Geological Survey, Western Ecological Research Center, Dixon Field Station, 800 Business Park Drive, Suite D, Dixon, California 95620 USA *pscoates@usgs.gov*

**S1: Spatial variable creation**

Vegetation was represented by the fractional components measured by Xian et al. (2015), including long-term averages of sagebrush and non-sagebrush cover percentages, perennial herbaceous vegetation percentages, shrub and sagebrush height, bare ground, and litter percentages at a 30-m pixel resolution. We characterized annual herbaceous grasses (e.g., *Bromus tectorum* or cheatgrass) on a yearly basis (Boyte & Wylie 2017; Boyte et al. 2019). We characterized pinyon-juniper coniferous cover using a similar high resolution map representing continuous percentages of pinyon-juniper (Gustafson et al. 2018). We incorporated normalized difference vegetation index (NDVI) to capture seasonal and long-term variation in greenness of vegetation (Pettorelli et al. 2011). Topography was characterized using indices developed from a digital elevation model (DEM) and the Geomorphology and Gradient Metrics Toolbox for ArcGIS™ (Evans et al. 2014). These included the compound topographic index (CTI), a steady state wetness index (Moore et al. 1993, Gessler et al. 1995), the topographic radiation aspect index (TRASP), a transformed aspect with the highest values (i.e., closest to one) identifying southwestern slopes (Stage 1976; Roberts & Cooper 1989), topographic roughness, a measure of terrain complexity, curvature, a measure of concavity/convexity (Bolstad & Lillesand 1992), and slope (Berry 2002). Hydrographic features were obtained from the National Hydrography Dataset (U.S. Geological Survey 2017). We also considered anthropogenic features, including roads (U.S. Census Bureau 2015) and developed areas (U.S. Census Bureau 2015). Cumulative burned area (CBA) for each year of the study through 2015 was estimated from the Monitoring Trends in Burned Severity dataset (Eidenshink et al. 2007; Coates et al. 2016a). We included resilience to disturbance and resistance to invasion (Chambers et al. 2014, Maestas et al 2016, Chambers et al. 2019). This layer is derived from soil moisture and temperature regimes along elevational gradients and classifies the relative productivity of a site and the ability of the site to maintain its historic state following disturbance (high, moderate, and low). This layer can be used to assess the relative risk of a site to transition to a novel state following disturbance such as wildfire, with low resilience and resistance classes being most likely to transition.

Using neighborhood analysis, we evaluated each metric within a circular moving window with radii of 75 m (1.8 ha), 167.9 m (8.7 ha), 439.5 m (61.5 ha), and 1,451.7 m (661.4 ha). These values represent the immediate area around the nest, the minimum, mean, and maximum daily distances traveled by sage-grouse (Coates et al. 2016b). In addition, we calculated Euclidean distances to line or point features (e.g., roads, streams, wet meadows). Distance metrics were ultimately transformed using an exponential decay function, $exp(-d/\propto)$, where *d* represented all distances and$\alpha$was specified as the average distance from the feature to the nest (Coates et al. 2016b).

Table S1: All landscape variables, metrics, scale of analysis (m), and source data used in nest selection and survival analyses.

| Type | Metric | Scales (m) | Sources |
| --- | --- | --- | --- |
| *Vegetation & Land Cover* | | | |
| Bare ground | 1 | Radii | Xian et al. 2015^1^ |
| Litter cover | 1 | Radii | Xian et al. 2015^1^ |
| Annual grass cover | 1 | Radii | Xian et al. 2015^1^ |
| Perennial grass cover | 1 | Radii | Xian et al. 2015^1^ |
| Big sagebrush | 1 | Radii | Xian et al. 2015^1^ |
| Low sagebrush | 1 | Radii | Xian et al. 2015^1^ |
| Total sagebrush cover | 1 | Radii | Xian et al. 2015^1^ |
| Non-sagebrush shrub canopy cover | 1 | Radii | Xian et al. 2015^1^ |
| Total shrub cover | 1 | Radii | Xian et al. 2015^1^ |
| Sagebrush shrub heights | 2 | Radii | Xian et al. 2015^1^ |
| Non-sagebrush shrub heights | 2 | Radii | Xian et al. 2015^1^ |
| Forests | 1 | Radii | Rollins 2009^2^; Homer et al. 2015^1^ |
| Distance to forest | 4 | Exponential decay | Rollins 2009^2^; Homer et al. 2015^1^ |
| Cropland | 2 | Radii | Rollins 2009^2^; Homer et al. 2015^1^ |
| Distance to cropland | 4 | Exponential decay | Rollins 2009^2^; Homer et al. 2015^1^ |
| Wet meadows | 1 | Radii | Rollins 2009^2^; Homer et al. 2015^1^ |
| Distance to wet meadow | 4 | Exponential decay | Rollins 2009^2^; Homer et al. 2015^1^ |
| Pinyon-juniper canopy cover | 1 | Radii | Coates et al. 2018^3^, Gustafson et al. 2018^3^ |
| Distance to pinyon-juniper cover class 1 | 4 | Exponential decay | Coates et al. 2018^3^, Gustafson et al. 2018^3^ |
| Distance to pinyon-juniper cover class 2 | 4 | Exponential decay | Coates et al. 2018^3^, Gustafson et al. 2018^3^ |
| Distance to nearest pinyon-juniper tree | 4 | Exponential decay | Coates et al. 2018^3^, Gustafson et al. 2018^3^ |
| Cheatgrass | 1 | Radii | Boyte & Wylie 2017; Boyte et al. 2019^4^ |
| Normalized Differentiated Vegetation Index (NDVI) | 3, 8 | 250, 439.5, 1451.9 | Didan 2015 |
| *Topography* | | | |
| Elevation | 3 | Radii | Digital Elevation Model (DEM)^5^ |
| Topographic roughness | 3 | Radii | DEM^5^, Evans et al. 2014^6^ |
| Slope | 3 | Radii | DEM^5^ |
| Curvature | 3 | Radii | DEM^5^ |
| Compound Topographic Index (CTI) | 3 | Radii | DEM^5^, Evans et al. 2014^6^ |
| *Anthropogenic development* | | | |
| All roads density | 5 | Radii | U.S. Census Bureau 2015^8^ |
| Distance to nearest road | 4 | Exponential decay | U.S. Census Bureau 2015^8^ |
| Small road density | 5 | Radii | U.S. Census Bureau 2015^8^ |
| Distance to nearest small road | 4 | Exponential decay | U.S. Census Bureau 2015^8^ |
| Primary or secondary road density | 5 | Radii | U.S. Census Bureau 2015^8^ |
| Distance to primary or secondary road | 4 | Exponential decay | U.S. Census Bureau 2015^8^ |
| Distance to powerline | 4 | Exponential decay | Platts^9^ |
| *Productivity & Disturbance* | | | |
| Cumulative Burned Area (CBA) | 3, 7 | 167.9, 439.5, 1451.7 | Eidenshink et al. 2007^10^ |
| Distance to Cumulative Burned Area (CBA) | 4, 7 | Exponential decay | Eidenshink et al. 2007^10^ |
| Resistance and Resilience | 3, 9 | 21.58 km | Maestas et al. 2016 |

1https://www.mrlc.gov/data

2https://www.landfire.gov/

3https://www.sciencebase.gov/catalog/item/59160b60e4b044b359e32e67

^4^<https://catalog.data.gov/dataset/a-time-series-of-herbaceous-annual-cover-in-the-sagebrush-ecosystem>

5https://viewer.nationalmap.gov/basic/

6https://evansmurphy.wixsite.com/evansspatial/arcgis-gradient-metrics-toolbox

7https://www.usgs.gov/core-science-systems/ngp/national-hydrography

8https://www.census.gov/geo/maps-data/data/tiger-line.html

9https://www.spglobal.com/platts/en/products-services/electric-power

10https://www.mtbs.gov/

Metric Legend:

1 = For each scale, mean value of a continuous percent cover for that vegetation type

2 = For each scale, proportion of pixels classified as dominant cover type. For each cover type, pixel was classified as either dominant (1) or not (0)

3 = For each scale, mean value of a continuous variable

4 = Transformed distances to linear or point features using exponential decay function

5 = Density of linear features (length/area), quantified in km × km^2^

6 = Density of point features (count/area), quantified in n × km^2^

7 = Cumulative value (time-dependent), incorporating recovery times specific to sagebrush RR class; pixel value = 1 if area burned without recovery to 20% sagebrush cover; 0 otherwise

8 = NDVI is a spatio-temporal variable, measured at 16-day intervals. We used a long-term mean pixel value across all breeding seasons

9 = Developed three numeric classes of relative soil temperature and moisture (1–3), where 1 = warm and dry conditions (i.e. low resilience and resistance), and 3 = cool and moist conditions (i.e. high resilience and resistance). Summed three numeric classifications to obtain a relative index across 22 km to account for regional variation in RR.

*Literature Cited*

Berry, JK (2002) Beyond mapping use surface area for realistic calculations. Geo World 15:20–21.

Bolstad PV, Lillesand TM (1992) Improved classification of forest vegetation in northern Wisconsin through a rule-based combination of soils, terrain, and Landsat Thematic Mapper data. Forest Science 38:5–20.

Chambers JC, Bradley BA, Brown CS, D'Antonio C, Germino MJ, Grace JB, Hardegree SP, Miller RF, Pyke DA (2014) Resilience to stress and disturbance, and resistance to Bromus tectorum L. invasion in cold desert shrublands of western North America. Ecosystems 17:360–375

Chambers JC, Allen CR, Cushman SA (2019a) Operationalizing Ecological Resilience Concepts for Managing Species and Ecosystems at Risk. Frontiers in Ecology and Evolution 7:241

Coates PS, Brussee BE, Howe KB, Gustafson KB, Casazza ML, Delehanty DJ (2016b) Landscape characteristics and livestock presence influence common ravens: relevance to greater sage-grouse conservation. Ecosphere 7.

Coates P S, Ricca MA, Prochazka BG, Brooks ML, Doherty KE, Kroger T, Blomberg EJ, Hagen CA, Casazza ML (2016a) Wildfire, climate, and invasive grass interactions negatively impact an indicator species by reshaping sagebrush ecosystems. Proceedings of the National Academy of Sciences of the United States of America 113:12745–12750.

Coates PS, Casazza ML, Ricca MA, Brussee BE, Blomberg EJ, Gustafson KB, Overton CT, Davis DM, Niell LE, Espinosa SP, Gardner SC, Delehanty DJ. (2016b) Integrating spatially explicit indices of abundance and habitat quality: an applied example for greater sage-grouse management. Journal of Applied Ecology 53:83–95

Didan, K. (2015). *MOD13Q1 MODIS/Terra Vegetation Indices 16-Day L3 Global 250m SIN Grid V006* [NDVI]. NASA EOSDIS Land Processes DAAC.

Eidenshink J, Schwind B, Brewer K, Zhu Z, Quayle B, Howard S (2007) A project for monitoring trends in burn severity. Fire Ecology 3: 3-21. Fire Ecology Special Issue Vol 3:4.

Evans J S, Oakleaf J, Cushman SA, and Theobald D (2014) An ArcGIS Toolbox for Surface Gradient and Geomorphometric Modeling, version 2.0-0. http://evansmurphy.wixsite.com/evansspatial/arcgis-gradient-metrics-toolbox.

Gustafson KB, Coates PS, Roth CL, Chenaille MP, Ricca MA, Sanchez-Chopitea E, Casazza ML (2018) Using object-based image analysis to conduct high-resolution conifer extraction at regional spatial scales. International Journal of Applied Earth Observation and Geoinformation 73:148–155.

Homer CG, Dewitz JA, Yang L, Jin S, Danielson P, Xian G, Coulston J, Herold ND, Wickham JD, Megown K (2015) Completion of the 2011 National Land Cover Database for the conterminous United States-Representing a decade of land cover change information. Photogrammetric Engineering and Remote Sensing 81:345–354.

Maestas JD, Campbell SB, Chambers JC, Pellant M, Miller RF (2016) Tapping soil survey information for rapid assessment of sagebrush ecosystem resilience and resistance. Rangelands 38:120–128.

Pettorelli N, Ryan S, Mueller T, Bunnefeld N, Jędrzejewska B, Lima M, Kausrud K (2011) The Normalized Difference Vegetation Index (NDVI): unforeseen successes in animal ecology. Climate Research 46:15–27.

Roberts DW, Cooper SV (1989) Concepts and techniques of vegetation mapping. General Technical Report INT-US Department of Agriculture, Forest Service, Intermountain Research Station (USA).

Rollins MG (2009) LANDFIRE: a nationally consistent vegetation, wildland fire, and fuel assessment. International Journal of Wildland Fire 18:235–249.

Stage AR (1976) An expression for the effect of aspect, slope, and habitat type on tree growth. Forest Science 22:457–460.

U.S. Census Bureau (2015) TIGER/Line Shapefiles (machine-readable data files). https://www.census.gov/geo/maps-data/data/tiger-line.html.

U.S. Geological Survey (2017) National Hydrography Dataset. https://nhd.usgs.gov.

Xian G, Homer C, Rigge M, Shi H, Meyer D (2015) Characterization of shrubland ecosystem components as continuous fields in the northwest United States. Remote Sensing of Environment 168:286–300.

**S2: Preliminary variable reduction**

To better elucidate functional relationships between landscape features and sage-grouse nest selection survival, we characterized habitat with multiple predictors, including nested scales and distance metrics. For example, we characterized road density within a range of scales meant to capture immediate conditions around the nest bowl (75 m) out to the maximum daily movement distance (1,451.7 m), as well as calculating the distance from nest to roads. However, this method has the potential to introduce collinearity. Specifically correlations (|*r|* > 0.6; Dormann et al. 2013) existed among similar representations of landscape predictors, and including too many predictors would result in overfitting (Harrell 2015). To avoid potential issues from multicollinearity and overfitting, we implemented a preliminary iterative variable reduction procedure designed to select only the most influential predictors of nest selection or survival among similar, correlated predictors, while also accounting for the influences of other uncorrelated predictors. We identified a unique set of candidate variables for both nest selection and survival models. We iteratively screened predictors within each suite of candidate variables for nest selection and survival within a loop of length *M* = 5000. Each iteration consisted of the following process, where **X** denotes the matrix of all candidate landscape predictors and *j* indexes the current iteration in 1:*M*:

1. We computed the correlation matrix of **X**_all_ and then used it to randomly generate a new matrix **X**_sub_ of uncorrelated predictors (all |*r*| < 0.6). This was done by first randomly selecting any predictor *x*_1_ from **X**_all_ and then randomly adding additional predictors *x*_i_ to **X**_sub_ under the condition that corr(*x*_i_, [*x*_1,…,_ *x*_k_]) < 0.6.
2. We began iteration *j* by fitting a model with 3-6 predictors randomly selected from **X**sub (current matrix = **X***j*). This was done to reduce the influence of potential model misspecification by omitting important predictors while avoiding overfitting from including too many predictors.
3. We fit a generalized linear mixed model relating the current set of predictors, **X***_j_*, to the binary response variable *Y* representing either a) presence/background location for the nest (nest site selection), or b) success/failure of the nest (nest survival).
   1. a) For nest site selection, the GLM was akin to a logistic regression model, where *Y ~ Bernoulli(p)* and the model was fit using the binomial family and a logit link function. We included a distance to lek variable in all models to account for clustering of nest locations near leks.
4. b) For nest survival, we used a logistic exposure model (Shaffer 2004), where *Y ~ Bernoulli(p)*, but instead of the logistic link we used a log-exposure link function (exposure = number of days that the nest was actively monitored; Bolker 2014). We included day of season and sage-grouse age effects for all models. We added a random intercept for year in the nest survival model to account for inter-annual variation. We temporarily stored the results of the model fit in (3), namely the AIC value of that model, e.g. ***baseline model [k]***. We also calculated variable importance for each functional form (i.e., linear, non-linear (2^nd^ order) and interaction with RR) for predictor *k* in model *j* as following:
   1. For each predictor *k*, we computed the AIC of a new model *without* that predictor and temporarily stored the AIC value for that model, e.g. ***removal model [-k]***.
   2. For each predictor *k* (excluding distance variables), we additionally considered non-linear (2^nd^ order terms) terms (i.e., *k + k^2^*). We calculated the difference (*d*AIC) between AIC(model *j*) and AIC(model *j* with the addition of *k^2^*), where a positive value indicated improvement in model fit when *k^2^* was included. If *d*AIC >2, indicating importance for the non-linear term, we temporarily stored AIC(model *j* with the addition of *k^2^*), e.g. ***quadratic model [k +k^2^]***.
   3. Finally, for each predictor *k* (excluding anthropogenic variables such as roads which are not expected to be influenced by RR), we considered a new model including an interaction term with RR (*k**RR). We calculated *d*AIC between AIC(model *j* including only the main effect for RR) and AIC(model *j* with the addition of *k**RR), where a positive value indicated improvement in model fit when *k**RR was included as opposed to only RR. If *d*AIC >2, indicating importance for the interaction term, we temporarily stored AIC(model *j* with the addition of *k**RR), e.g. ***interaction model [k*RR]***.
   4. We then tracked which model had the lowest AIC (i.e. baseline model, removal model, quadratic model, or interaction model), and we saved *d*AIC between AIC(best model) and AIC (model *j* without *k*), which represents the total contribution of the best functional form for predictor *k*.

5. At the conclusion of the loop, for each predictor, we summarized which functional form was most frequently ranked as “best”. Additionally, we averaged across all *d*AIC scores of top models for each predictor and ranked predictors by their average *d*AIC across all models that included them. For correlated predictors, only the predictor with the greatest dAIC score was considered in subsequent models.

*Literature Cited*

Bolker BM (2014) Logistic regression, accounting for differences in exposure. https://rpubs.com/bbolker/logregexp.

Dormann CF, Elith J, Bacher S, Buchmann C, Carl G, Carre G, Garcia Marquez JR, Gruber B, Lafoourcade B, Leitao PJ, Münkemüller T, Mcclean C, Osborne PE, Reineking B, Schröder B, Skidmore AK, Zurell D  Lautenbach S (2013) Collinearity: a review of methods to deal with it and a simulation study evaluating their performance. Ecography 36:27–46

Harrell FE (2015) Multivariable modeling strategies. In: Regression modeling strategies. Springer pp. 63–102.

Shaffer TL (2004) A unified approach to analyzing nest success. Auk 121:526–540.

**S3: modeling and mapping pre-fire nest site selection**

*Random locations generation*

Because sage-grouse nest locations are likely to occur near leks (Holloran & Anderson 2005; Coates et al. 2013), we conditioned random locations based on the distribution of active leks at the study site. Specifically, we first subsampled leks within 20 km of any nest located at the site to delineate a maximum boundary for the local population. Then, we calculated proximity to nearest lek for each nest. We used the maximum resulting distance (17 km) to buffer around all leks. We combined the buffers and generated 10 random locations per nest within the overall buffered area. We also incorporated distance to lek in the nest RSF described above to account for clustering of nest locations near leks (i.e., the frequency of nest locations is a decaying function of distance to lek; Coates et al. 2013). The choice of a 10:1 ratio of random to used locations was intended to appropriately weight the available distribution (e.g., maximizing the number of available locations; Northrup et al. 2013, Fieberg et al. 2021) without oversaturating the locations within the study’s geographic extent.

*Nest site selection RSF*

Prior to modeling nest selection, we standardized all habitat metrics so that their mean was 0 and standard deviation was 1. To promote model precision and further protect against potential overfitting, we specified shrinkage prior distributions (Authier et al. 2017) for the habitat covariates according to the Bayesian lasso (Park & Casella 2008), where the vector of coefficients, **β**_RSF_ = *β*_1_,…, *β_k_*, within the nest RSF each followed the Laplace distribution, β ~ *Laplace* (0,λ), where λ ~ U(0,10). The Laplace distribution is also referred to as the double exponential. The prior for the Laplace scale parameter was uninformative, such that a value of 0 would implement a ridge regression (similar predictor coefficients shrunk towards one another), whereas 10 would indicate a lasso implementation (Friedman et al. 2010, Tibshirani et al. 2012). In the resulting multivariate model, coefficients that have minimal support from the data are shrunk towards zero and have approximately zero influence on the overall linear predictor (Authier et al. 2017). Random effects priors were uninformative, where 0.0001 was precision (1/σ^2^). We ran 3 chains of 50,000 iterations after a burn-in of 10,000, thinning by a factor of 10. We examined chains and calculated Gelman-Rubin statistics ($\hat{r}$<1.05) to verify convergence of all parameters. We reported the results of all coefficient estimates (i.e. fixed effects) in terms of median posterior values and 2.5^th^ and 97.5^th^ percentiles (95% CRI) but limited our interpretations to those with 95% posterior CRI not overlapping zero.

*Mapping relative nest site selection*

We used the estimates for each spatially explicit landscape covariate from the final pre-fire nest selection RSF model to generate a predicted surface of relative site selection within our study area. The nest RSF was modeled as:

$$\hat{w}(x)=\exp(\beta_{1}x_{1}+\beta_{2}x_{2}+...+\beta_{k}x_{k})$$

where each *β* represents a coefficient estimate for each predictor (*x_1_*,…,*x_k_*) (Johnson et al. 2006, McDonald 2013). In this case, the random intercepts for sage-grouse and year were omitted, as relative selection is the same across individuals and years due to sampling design. If temporally-varying covariates such as NDVI or annual grass were informative, we calculated a long-term average raster (years 2009–2016) for these covariates when making the pre-fire prediction. We applied this function to all pixels within the study area extent to map the pre-fire nest site RSF. We rescaled $\hat{w}(x)$ between 0 and 1 to obtain a stretched habitat selection score following Johnson (2004) and Ricca et al. (2018):

$$\hat{w}=(w[x]-w_{\min})/(w_{\max}-w_{\min})$$

where each pixel value (i.e., *w*[*x*]) is scaled relative to the overall minimum and maximum value of the pre fire RSF values.

##############################################################################

#The following R code provides the nest resource selection model specification for implementation using

#JAGS language, given the data provided. The code requires local installation of JAGS #software as well as the jagsUI library.

##############################################################################

library(spatial)

library(sp)

library(jagsUI)

library(raster)

library(rgdal)

jags.data = dget("data/nestSel_data.R") #example input data file

modelFilename = "code/NestSel_vmFoD.txt"

cat("

model{

### Priors ###

sd.year ~ dunif(0,100)

sd.bird ~ dunif(0,100)

for (j in 1:Nyear) {

kappa.year[j] ~ dnorm(0, tau.year)

}

for (j in 1:Nbird) {

kappa.bird[j] ~ dnorm(0, tau.bird)

}

tau.year = 1/pow(sd.year,2)

tau.bird = 1/pow(sd.bird,2)

b0 ~ dnorm(0,0.0001)

### Indicator variable selection for Beta

for (k in 1:n.pred) {

beta[k] ~ ddexp(0, lambda)

}

lambda ~ dunif(0,10) # lambda --> 0 = ridge regression; lambda --> 10 = LASSO

### Likelihood

for (i in 1:n.row) {

y[i] ~ dbern(psi[i])

logit(psi[i]) <- b0 + inprod(X[i,],beta) + kappa.year[year[i]] + kappa.bird[bird[i]]

}

}

", fill=TRUE, file=modelFilename)

### INITIAL VALUES ###

inits = function() {

list(b0=runif(1),

beta=runif(jags.data$n.pred,-1,1),

kappa.year = rep(0,max(jags.data$year)),

kappa.bird = rep(0,max(jags.data$bird)))

}

### PARAMETERS ###

params = c("b0","beta","kappa.year","kappa.bird")

out.model = jags(jags.data, inits, params, modelFilename,

n.thin=10, n.chains=3, n.adapt=10000, n.iter=50000,

parallel=TRUE, n.cores=3)

*Literature Cited*

Authier M, Saraux C, Péron C (2017) Variable selection and accurate predictions in habitat modelling: a shrinkage approach. Ecography 40:549–560

Coates PS, Casazza ML, Blomberg EJ, Gardner SC, Espinosa SP, Yee JL, Wiechman L, Halstead BJ (2013) Evaluating greater sage-grouse seasonal space use relative to leks: Implications for surface use designations in sagebrush ecosystems. J. Wildlife Manage*.* 77: 1598–1609

Fieberg, J, Signer, J, Smith, B, Avgar, TA (2021) A ‘How to’ guide for interpreting parameters in habitat selection analyses. Journal of Animal Ecology 90: 1027–1043.

Friedman J, Hastie T, Tibshirani R (2010) Regularization paths for generalized linear models via coordinate descent. Journal of statistical software 33:1

Holloran MJ, Anderson SH (2005) Spatial distribution of greater sage-grouse nests in relatively contiguous sagebrush habitats. The Condor 107:742–752

Northrup, JM, Hooten, MN, Anderson Jr, CR, Wittemyer, G. (2013) Practical guidance on characterizing availability in resource selection functions under a use-availability design. Ecology 94:145­6­–1463.

Park T, Casella G (2008) The bayesian lasso. Journal of the American Statistical Association 103:681–686

Tibshirani R, Bien J, Friedman J, Hastie T, Simon N, Taylor J, Tibshirani RJ (2012) Strong rules for discarding predictors in lasso‐type problems. Journal of the Royal Statistical Society: Series B (Statistical Methodology) 74:245–266

**S4: modeling and mapping pre-fire nest survival**

In our nest survival model, survival was assumed to be a continuous process. The unit daily hazard was modeled as

$$UH_{hijk}=\exp(\gamma_{0}+\mathbf{X\beta}+\kappa_{j}+\tau_{k})$$

where *h,* *i,* *j*, and *k* refer to individual nest, day, year, and sage-grouse, respectively. Similar to the nest RSF, **Xβ** comprise the landscape habitat coefficients **β** multiplied by fixed effects covariates **X**. Landscape habitat coefficients were identified using a variable reduction process (Appendix S2, S3), and we also considered fixed effects for age of the sage-grouse (yearling vs. adult) and day of season for nest first found. Day of season was standardized relative to the overall mean for each year, and all habitat covariates were standardized around their means and standard deviations. As with RSFs, we incorporated random effects for year (κ*_j_*), and individual sage-grouse (τ*_k_*) to account for sage-grouse with multiple nests and repeated sampling within the same study site across years (Bolker et al. 2009; Gillies et al. 2006).

Like the nest RSF, we evaluated the nest frailty model using MCMC methods in JAGS (Plummer 2003; Kellner 2018). We assigned uninformative uniform priors to random effects, κ*_j_* ~ N(0,0.0001); τ*_k_* ~N(0,0.0001), and applied the Bayesian lasso prior to the coefficients of the nest frailty model, where the prior distributions of the habitat covariates are specified following the methods described for the nest RSF analysis (Appendix S3).

*Relative pre-fire nest survival map*

As with the pre-fire nest selection RSF, we mapped expected pre-fire nest survival based on the median posterior coefficient values from the nest frailty model (i.e., the posterior predictive distribution), where the cumulative 38-day nesting period (laying + incubation) hazard was

$$CH_{hijk}=\sum_{i=1}^{T=38} UH_{hijk}$$

Where *UH_hijk_* included only the relevant habitat coefficients (see Eq. 1) and expected nest success was then the cumulative daily rate across 38 days, expressed as

$$S_{hjk}=e^{-CH_{hjk}}$$

For mapping purposes, the random effect estimates for sage-grouse and year were set to 0 to predict a long-term average across sage-grouse and years. To capture variation in nest survival by age class, we generated separate estimates for yearlings and adults, and applied a weighted average of those estimates based on the proportion of yearlings vs. adults present in the dataset. As in the nest site RSF prediction, if temporally-varying spatial covariates were informative, we used long-term averages for temporally-varying spatial predictors. Similarly, day of season (i.e. approximate nest initiation date) was set to its average across years 2009–2016.

##############################################################################

#The following R code provides the nest frailty model specification for implementation using #JAGS language, given the data provided. The code requires local installation of JAGS #software as well as the jagsUI library.

##############################################################################

library(jagsUI)

jags.data = dget("data/nestSurv_data.R") #example input data file

### MODEL ###

modelFilename="code/nestSurv_vmFoD.txt"

cat("

model{

# PRIORS #

gamma.n~dunif(-20,0)

# RANDOM EFFECTS #

sd.year~dunif(0,100)

tau.year<-1/pow(sd.year,2)

for(j in 1:Nyear){ # loop for random draws of each year

kappa.year[j] ~ dnorm(0, tau.year)

}

sd.hen~dunif(0,100)

tau.hen<-pow(sd.hen,-2)

for(k in 1:n.hen){ # random draws of each hen (to account for several with multiple nests)

nu.hen[k]~dnorm(0,tau.hen)

}

# LIKELIHOOD #

for (k in 1:n.pred) {

w[k] ~ dbern(1) # prior inclusion probability

betaT[k] ~ ddexp(0,lambda) # Laplace double exponential prior

beta[k]<-w[k]*betaT[k] # switches beta[l] on/off (if w=0)

}

lambda ~ dunif(0,10) # lambda --> 0 = ridge regression; lambda --> 10 = LASSO

for(i in 1:n.row){

X[i,11]~dbern(0.87) # prior to fill in missing data on age of hen

for(j in left[i]:(right[i]-1)){ # loop from entry to exit

UH[i,j]<-exp(gamma.n + kappa.year[year[i]] + nu.hen[hen[i]] + inprod(X[i,],beta))

}

SR[i]<-min(exp(-sum(UH[i,left[i]:(right[i]-1)])), 0.99999) # exponentiate the negative of the cumulative sum of the unit hazard

censored[i]~dbern(max(SR[i], 0.00001)) # failed or censored? - Bernoulli trial with cumulative probability from previous line

A.ppd.save[i]<-SR[i] # Posterior predictive distribution for calculating WAIC

}

}

",fill=TRUE,file=modelFilename)

### Initial values ###

inits<-function(){

list(gamma.n=runif(1,-5,-2), sd.hen=runif(1,0,2), nu.hen=rep(0,jags.data$n.hen),

sd.year=runif(1,0,2), kappa.year=rep(0,jags.data$Nyear),

betaT=runif(jags.data$n.pred,-1,1), w=rbinom(jags.data$n.pred,1,0.5))}

### Parameters to Monitor ###

params<-c("gamma.n", "beta", "nu.hen","sd.hen","w",

"kappa.year","sd.year", "SR", "A.ppd.save")

out.model = jags(jags.data, inits, params, modelFilename,

n.thin=10, n.chains=3, n.adapt=10000, n.iter=50000,

parallel=TRUE, n.cores=3)

*Literature Cited*

Bolker BM, Brooks ME, Clark CJ, Geange SW, Poulsen JR, Stevens MHM, White JSJ (2009) Generalized linear mixed models: a practical guide for ecology and evolution. Trends in Ecology & Evolution 24:127–135

Gillies CS, Hebblewhite M, Nielson SE, Krawchuk MA, Aldridge CL, Frair JL, Saher DJ, Stevens CE, Jerde CL (2006) Application of random effects to the study of resource selection by animals. Journal of Animal Ecology 75:887–898

Johnson CJ, Seip DR, Boyce MS (2004) A quantitative approach to conservation planning: using resource selection functions to map the distribution of mountain caribou at multiple spatial scales. J Appl Ecol 41

Johnson CJ, Nielsen SE, Merrill EH, McDonald TL, Boyce MS (2006) Resource selection functions based on use–availability data: theoretical motivation and evaluation methods. Journal of Wildlife Management 70:347–357

Kellner K (2018) jagsUI: a wrapper around ‘rjags’ to streamline ‘JAGS’ analyses.

McDonald TL (2013) The point process use‐availability or presence‐only likelihood and comments on analysis. Journal of Animal Ecology 82:1174–1182

Plummer M (2003) JAGS: A program for analysis of Bayesian graphical models using Gibbs sampling. In: Proceedings of the 3rd international workshop on distributed statistical computing 124 Vienna, Austria p. 125.

Ricca MA, Coates PS, Gustafson KB, Brussee BE, Chambers JC, Espinosa SP, Gardner SC, Lisius S, Zielgler P, Delehandty DJ, Casazza ML (2018) A conservation planning tool for Greater Sage‐grouse using indices of species distribution, resilience, and resistance. Ecological applications 28:878–896

**S5: Code for sagebrush recovery simulations**

##############################################################################

#This code provides the workflow for developing sagebrush recovery simulations

#for passive, grazing exclusion, seeding, and seedling recovery scenarios

#to restore Greater sage-grouse habitat within the Virginia Mountains study site

#recovery is based on sagebrush community and ecological resilience to disturbance and resistance to invasion

##############################################################################

require(rgdal)

require(raster)

require(sp)

require(rgeos)

##############################################################################

#MAPPING SAGEBRUSH COMMUNITIES

##############################################################################

#Mapping sagebrush communities based on the Biophysical Potential Settings

#first, identify the biophysical potential settings for sagebrush Wyoming Big, Mountain Big, Low (dwarf and black)

#we used the BPS settings from Brooks et al. 2015 (Table 1). We were provided additional BPS settings in several tables. We have combined these into a single table within this supplemental material for user access (Table S2)

#read in full classification table (Table S2):

sage_table <- read.csv("Sagebrush_ESP.csv")

#identify the settings of interest on the raster product:

esp <- raster("Environmental_Site_Potential.tif") #the environmental site potential raster

rr <- raster("RR_Classes_Rangewide_30m.tif") #resilience to disturbance and resistance to invasion raster

esp <- crop(esp, rr)

sage <- subs(esp, sage_table[,c("VALUE", "Community")], subsWithNA = T)

writeRaster(sage, "Sagebrush_Biophysical_Potential_30m.tif")

#separate the three communities:

low <- sage

low[low != 1] <- NA

big <- sage

big[big != 2] <- NA

mtn <- sage

mtn[mtn != 3] <- NA

#separate R&R classes:

rr_1 <- rr

rr_1[rr_1 != 1] <- NA

rr_2 <- rr

rr_2[rr_2 != 2] <- NA

rr_3 <- rr

rr_3[rr_3 != 3] <- NA

##############################################################################

#PASSIVE SCENARIO

##############################################################################

#Creating an annual percent cover return surface for passive recovery:

low_1 <- mask(rr_1, low, inverse = T, updatevalue = 0.009)

low_1[low_1 == 1] <- NA #drop places where there was no sagebrush (rr1 = 1 rr2 = 2, rr3 = 3); for merge later

big_1 <- mask(rr_1, big, inverse = T, updatevalue = 0.0038)

big_1[big_1 == 1] <- NA

mtn_1 <- mask(rr_1, mtn, inverse = T, updatevalue = 0.024)

mtn_1[mtn_1 == 1] <- NA

rr_1_passive <- merge(low_1, big_1, mtn_1)

low_2 <- mask(rr_2, low, inverse = T, updatevalue = 0.005)

low_2[low_2 == 2] <- NA

big_2 <- mask(rr_2, big, inverse = T, updatevalue = 0.0015)

big_2[big_2 == 2] <- NA

mtn_2 <- mask(rr_2, mtn, inverse = T, updatevalue = 0.014)

mtn_2[mtn_2 == 2] <- NA

rr_2_passive <- merge(low_2, big_2, mtn_2)

low_3 <- mask(rr_3, low, inverse = T, updatevalue = 0.0025)

low_3[low_3 == 3] <- NA

big_3 <- mask(rr_3, big, inverse = T, updatevalue = 0.00075)

big_3[big_3 == 3] <- NA

mtn_3 <- mask(rr_3, mtn, inverse = T, updatevalue = 0.007)

mtn_3[mtn_3 == 3] <- NA

rr_3_passive <- merge(low_3, big_3, mtn_3)

rr_3_passive <- sampleRandom(rr_3_passive, (ncell(rr_3_passive) * 0.1), asRaster = T) #We are simulating a 10% success rate for R&R3

#combine them all into a single raster:

passive_rec <- merge(rr_1_passive, rr_2_passive, rr_3_passive)

#thirty year:

sage_30 <- passive_rec * 30

sage_30[sage_30 > 1] <- 1 #100% cover as high as we go

#fifty year:

sage_50 <- passive_rec * 50

sage_50[sage_50 > 1] <- 1 #100% cover as high as we go

#these layers were clipped to the Virginia Mountains study site in ArcMap

#modifying recovery based on probability of sagebrush establishment (passive):

pass_rec_30 <- raster("Passive_30yr_Recovered_VM.tif")

pass_est_30 <- pass_rec_30

pass_probs <- runif(ncell(pass_est_30), min = 0.10, max = 0.25) #establishment distribution

establish <- sapply(pass_probs, function(p){sample(c(TRUE, FALSE), 1, prob = c(p, 1 - p))}) #randomly sample from distribution

pass_est_30[] <- as.integer(establish) #assign the randomly sampled distribution to cells in the raster

pass_est_30[is.na(pass_rec_30)] <- 1 #retain original NAs

pass_est_30[pass_rec_30 == 0] <- 1 #retain original no recovery cells

#now remove the cells that don't germinate from the original raster

pass_rec_30[pass_est_30 == 0] <- NA

#calculate total recovered acres:

passive_acres_30 <- (ncell(pass_rec_30[pass_rec_30 >= 0.20]) * 900) * 0.000247105

#priority area:

pass_all_30 <- pass_rec_30

pass_all_30[pass_all_30 >= 0.20] <- 1

pass_all_30[pass_all_30 != 1] <- 0

dir.create("Established")

writeRaster(pass_all_30, "Established/Passive_30_Established.tif")

#multiply and keep priority areas:

#read in original post-fire recovery index layer:

rec.index <- raster("fire_restore_index.tif")

rec_index <- projectRaster(rec_index, pass_bin_30)

pass_pri_30 <- pass_bin_30 * rec_pri

pass_pri_30 <- round(pass_pri_30, 0)

passive_acres_priority_30 <- (ncell(pass_pri_30[pass_pri_30 >= 1]) * 900) * 0.000247105 #recovered within priority habitat

#reclassify, keeping only 3 and 4:

pass_pri_30[pass_pri_30 < 3] <- 0

#export:

dir.create("Prioritized_Recovery")

writeRaster(pass_pri_30, "Prioritized_Recovery/Passive_30yr_Priority.tif")

#50 years after fire

pass_rec_50 <- raster("Passive_50yr_Recovered_VM.tif")

pass_rec_50[pass_est_30 == 0] <- NA # remove the same cells that don't establish from the 50 yr raster

passive_acres_50 <- (ncell(pass_rec_50[pass_rec_50 >= 0.20]) * 900) * 0.000247105

pass_all_50 <- pass_rec_50

pass_all_50[pass_all_50 >= 0.20] <- 1

pass_all_50[pass_all_50 != 1] <- 0

writeRaster(pass_all_50, "Established/Passive_50_Established.tif", overwrite = T)

pass_pri_50 <- pass_all_50 * rec.index

pass_pri_50 <- round(pass_pri_50, 0)

passive_acres_priority_50 <- (ncell(pass_pri_50[pass_pri_50 >= 1]) * 900) * 0.000247105

pass_pri_50[pass_pri_50 < 3] <- 0

writeRaster(pass_pri_50, "Prioritized_Recovery/Passive_50yr_Priority.tif")

##############################################################################

#GRAZING SCENARIO

##############################################################################

#modifying recovery based on probability of sagebrush establishment (grazing):

graze_rec_30 <- raster("Passive_30yr_Recovered_VM.tif") #use passive recovery rates

graze_est_30 <- graze_rec_30

graze_probs <- runif(ncell(graze_est_30), min = 0.10, max = 0.25)

establish <- sapply(graze_probs, function(p){sample(c(TRUE, FALSE), 1, prob = c(p, 1 - p))})

#30 years after fire

graze_est_30[] <- as.integer(establish)

graze_est_30[is.na(graze_rec_30)] <- 1

graze_est_30[graze_rec_30 == 0] <- 1

graze_rec_30[graze_est_30 == 0] <- NA

grazing_acres_30 <- (ncell(graze_rec_30[graze_rec_30 >= 0.20]) * 900) * 0.000247105

graze_all_30 <- graze_rec_30

graze_all_30[graze_all_30 >= 0.20] <- 1

graze_all_30[graze_all_30 != 1] <- 0

writeRaster(graze_all_30, "Established/Grazing_30yr_Established.tif")

graze_pri_30 <- graze_bin_30 * rec_pri

graze_pri_30 <- round(graze_pri_30, 0)

grazing_acres_priority_30 <- (ncell(graze_pri_30[graze_pri_30 >= 1]) * 900) * 0.000247105

graze_pri_30[graze_pri_30 < 3] <- 0

writeRaster(graze_pri_30, "Prioritized_Recovery/Grazing_30yr_Priority.tif")

#50 years after fire

graze_rec_50 <- raster("Passive_50yr_Recovered_VM.tif")

graze_rec_50[graze_est_30 == 0] <- NA

grazing_acres_50 <- (ncell(graze_rec_50[graze_rec_50 >= 0.20]) * 900) * 0.000247105

graze_all_50 <- graze_rec_50

graze_all_50[graze_all_50 >= 0.20] <- 1

graze_all_50[graze_all_50 != 1] <- 0

writeRaster(graze_all_50, "Established/Grazing_50yr_Established.tif", overwrite = T)

graze_pri_50 <- graze_all_50 * rec.index

graze_pri_50 <- round(graze_pri_50, 0)

grazing_acres_priority_50 <- (ncell(graze_pri_50[graze_pri_50 >= 1]) * 900) * 0.000247105

graze_pri_50[graze_pri_50 < 3] <- 0

writeRaster(graze_pri_50, "Prioritized_Recovery/Grazing_50yr_Priority.tif")

##############################################################################

#SEEDING SCENARIO

##############################################################################

#Creating an annual percent cover return surface for seeding recovery:

#note slight change in code, it just goes faster this way but I wanted to preserve the individual SB communities in the passive script

rr_1_seed <- mask(rr_1, low, inverse = T, updatevalue = 0.0098)

rr_1_seed <- mask(rr_1_seed, big, inverse = T, updatevalue = 0.0039)

rr_1_seed <- mask(rr_1_seed, mtn, inverse = T, updatevalue = 0.027)

rr_2_seed <- mask(rr_2, low, inverse = T, updatevalue = 0.0054)

rr_2_seed <- mask(rr_2_seed, big, inverse = T, updatevalue = 0.0016)

rr_2_seed <- mask(rr_2_seed, mtn, inverse = T, updatevalue = 0.015)

rr_3_seed <- mask(rr_3, low, inverse = T, updatevalue = 0.0027)

rr_3_seed <- mask(rr_3_seed, big, inverse = T, updatevalue = 0.0008)

rr_3_seed <- mask(rr_3_seed, mtn, inverse = T, updatevalue = 0.0075)

rr_3_seed <- sampleRandom(rr_3_seed, (ncell(rr_3_seed) * 0.9), asRaster = T) #We are simulating a 90% success rate for seeding in RR3

seed_rec <- merge(rr_1_seed, rr_2_seed, rr_3_seed)

seed_rec[seed_rec == 0] <- NA

seed_rec[seed_rec >= 1] <- NA

seed_30 <- seed_rec * 30

seed_30[seed_30 > 1] <- 1

seed_50 <- seed_rec * 50

seed_50[seed_50 > 1] <- 1

#these layers were clipped to the Virginia Mountains study site in ArcMap

#modifying recovery based on probability of sagebrush establishment (seeding):

seed_rec_30 <- raster("Seeding_30yr_Recovered_VM.tif")

seed_est_30 <- seed_rec_30

seed_probs <- runif(ncell(seed_est_30), min = 0.25, max = 0.50)

establish <- sapply(seed_probs, function(p){sample(c(TRUE, FALSE), 1, prob = c(p, 1 - p))})

#30 years after fire

seed_est_30[] <- as.integer(establish)

seed_est_30[is.na(seed_rec_30)] <- 1

seed_est_30[seed_rec_30 == 0] <- 1

seed_rec_30[seed_est_30 == 0] <- NA

seeding_acres_30 <- (ncell(seed_rec_30[seed_rec_30 >= 0.20]) * 900) * 0.000247105

seed_all_30 <- seed_rec_30

seed_all_30[seed_all_30 >= 0.20] <- 1

seed_all_30[seed_all_30 != 1] <- 0

writeRaster(seed_all_30, "Established/Seeding_30yr_Established.tif")

seed_pri_30 <- seed_bin_30 * rec_pri

seed_pri_30 <- round(seed_pri_30, 0)

seeding_acres_priority_30 <- (ncell(seed_pri_30[seed_pri_30 >= 1]) * 900) * 0.000247105

seed_pri_30[seed_pri_30 < 3] <- 0

writeRaster(seed_pri_30, "Prioritized_Recovery/Seeding_30yr_Priority.tif")

#50 years after fire

seed_rec_50 <- raster("Seeding_50yr_Recovered_VM.tif")

seed_rec_50[seed_est_30 == 0] <- NA

seeding_acres_50 <- (ncell(seed_rec_50[seed_rec_50 >= 0.20]) * 900) * 0.000247105

seed_all_50 <- seed_rec_50

seed_all_50[seed_all_50 >= 0.20] <- 1

seed_all_50[seed_all_50 != 1] <- 0

writeRaster(seed_all_50, "Established/Seeding_50yr_Established.tif", overwrite = T)

seed_pri_50 <- seed_all_50 * rec.index

seed_pri_50 <- round(seed_pri_50, 0)

seeding_acres_priority_50 <- (ncell(seed_pri_50[seed_pri_50 >= 1]) * 900) * 0.000247105

seed_pri_50[seed_pri_50 < 3] <- 0

writeRaster(seed_pri_50, "Prioritized_Recovery/Seeding_50yr_Priority.tif")

##############################################################################

#PLANTING SCENARIO

##############################################################################

#Creating an annual percent cover return surface for seedling recovery:

rr_1_plant <- mask(rr_1, low, inverse = T, updatevalue = 0.014)

rr_1_plant <- mask(rr_1_plant, big, inverse = T, updatevalue = 0.0097)

rr_1_plant <- mask(rr_1_plant, mtn, inverse = T, updatevalue = 0.038)

rr_2_plant <- mask(rr_2, low, inverse = T, updatevalue = 0.0082)

rr_2_plant <- mask(rr_2_plant, big, inverse = T, updatevalue = 0.0026)

rr_2_plant <- mask(rr_2_plant, mtn, inverse = T, updatevalue = 0.022)

rr_3_plant <- mask(rr_3, low, inverse = T, updatevalue = 0.0041)

rr_3_plant <- mask(rr_3_plant, big, inverse = T, updatevalue = 0.0013)

rr_3_plant <- mask(rr_3_plant, mtn, inverse = T, updatevalue = 0.011)

rr_3_plant <- sampleRandom(rr_3_plant, (ncell(rr_3_plant) * 0.9), asRaster = T) #We are simulating a 90% success rate for planting in RR3

plant_rec <- merge(rr_1_plant, rr_2_plant, rr_3_plant)

plant_rec[plant_rec == 0] <- NA

plant_rec[plant_rec >= 1] <- NA

plant_30 <- plant_rec * 30

plant_30[plant_30 > 1] <- 1

plant_50 <- plant_rec * 50

plant_50[plant_50 > 1] <- 1

#these layers were clipped to the Virginia Mountains study site in ArcMap

###Modifying recovery based on probability of sagebrush establishment (planting):

plant_rec_30 <- raster("Planting_30yr_Recovered_VM.tif")

plant_est_30 <- plant_rec_30

plant_probs <- runif(ncell(plant_est_30), min = 0.50, max = 1)

establish <- sapply(plant_probs, function(p){sample(c(TRUE, FALSE), 1, prob = c(p, 1 - p))})

#30 years after fire:

plant_est_30[] <- as.integer(establish)

plant_est_30[is.na(plant_rec_30)] <- 1

plant_est_30[plant_rec_30 == 0] <- 1

plant_rec_30[plant_est_30 == 0] <- NA

planting_acres_30 <- (ncell(plant_rec_30[plant_rec_30 >= 0.20]) * 900) * 0.000247105

plant_all_30 <- plant_rec_30

plant_all_30[plant_all_30 >= 0.20] <- 1

plant_all_30[plant_all_30 != 1] <- 0

writeRaster(plant_all_30, "Established/Planting_30yr_Established.tif")

plant_pri_30 <- plant_bin_30 * rec_pri

plant_pri_30 <- round(plant_pri_30, 0)

planting_acres_priority_30 <- (ncell(plant_pri_30[plant_pri_30 >= 1]) * 900) * 0.000247105

plant_pri_30[plant_pri_30 < 3] <- 0

writeRaster(plant_pri_30, "Prioritized_Recovery/Planting_30yr_Priority.tif")

#50 years after fire

plant_rec_50 <- raster("Planting_50yr_Recovered_VM.tif")

plant_rec_50[plant_est_30 == 0] <- NA

planting_acres_50 <- (ncell(plant_rec_50[plant_rec_50 >= 0.20]) * 900) * 0.000247105

plant_all_50 <- plant_rec_50

plant_all_50[plant_all_50 >= 0.20] <- 1

plant_all_50[plant_all_50 != 1] <- 0

plant_pri_50 <- plant_all_50 * rec.index

plant_pri_50 <- round(plant_pri_50, 0)

planting_acres_priority_50 <- (ncell(plant_pri_50[plant_pri_50 >= 1]) * 900) * 0.000247105

plant_pri_50[plant_pri_50 < 3] <- 0

| Table S2: biophysical potential settings that were used to identify sagebrush communities across the western U.S. Data are derived from LANDFIRE succession modeling. Terms are defined in the FEIS Glossary (http://www.fs.fed.us/database/feis/glossary2.html). | | | |
| --- | --- | --- | --- |
| Region | Biophysical Setting name | BpS code | Community |
| California | Inter-Mountain Basins montane sagebrush steppe | 611260 | Mountain |
| Great Basin | Inter-Mountain Basins montane sagebrush steppe | 1211260 | Mountain |
| Great Basin | Inter-Mountain Basins montane sagebrush steppe | 1311260 | Mountain |
| Great Basin | Inter-Mountain Basins montane sagebrush steppe | 1711260 | Mountain |
| Great Basin | Inter-Mountain Basins montane sagebrush steppe | 1811260 | Mountain |
| Great Basin | Inter-Mountain Basins montane sagebrush steppe - mountain big sagebrush | 1611261 | Mountain |
| Northern and Central Rockies | Inter-Mountain Basins montane sagebrush steppe | 1011260 | Mountain |
| Northern and Central Rockies | Inter-Mountain Basins montane sagebrush steppe | 1911260 | Mountain |
| Northern and Central Rockies | Inter-Mountain Basins montane sagebrush steppe | 2011260 | Mountain |
| Northern and Central Rockies | Inter-Mountain Basins montane sagebrush steppe | 2111260 | Mountain |
| Northern and Central Rockies | Inter-Mountain Basins montane sagebrush steppe | 2211260 | Mountain |
| Northern and Central Rockies | Inter-Mountain Basins montane sagebrush steppe | 2911260 | Mountain |
| Northern Great Plains | Inter-Mountain Basins big sagebrush shrubland | 3310800 | Mountain |
| Northern Great Plains | Inter-Mountain Basins montane sagebrush steppe | 3311260 | Mountain |
| Pacific Northwest | Inter-Mountain Basins montane sagebrush steppe | 111260 | Mountain |
| Pacific Northwest | Inter-Mountain Basins montane sagebrush steppe | 711260 | Mountain |
| Pacific Northwest | Inter-Mountain Basins montane sagebrush steppe | 811260 | Mountain |
| Pacific Northwest | Inter-Mountain Basins montane sagebrush steppe | 911260 | Mountain |
| South-Central US | Inter-Mountain Basins montane sagebrush steppe | 2611260 | Mountain |
| Southwest | Inter-Mountain Basins big sagebrush shrubland | 1510800 | Mountain |
| Southwest | Inter-Mountain Basins big sagebrush shrubland | 2810800 | Mountain |
| Southwest | Inter-Mountain Basins montane sagebrush steppe | 2511260 | Mountain |
| Southwest | Inter-Mountain Basins montane sagebrush steppe | 2711260 | Mountain |
| Southwest | Inter-Mountain Basins montane sagebrush steppe | 2811260 | Mountain |
| Southwest | Inter-Mountain Basins montane sagebrush steppe - mountain big sagebrush | 2311261 | Mountain |
| Southwest | Inter-Mountain Basins montane sagebrush steppe - mountain big sagebrush | 2411261 | Mountain |
| California | Inter-Mountain Basins big sagebrush shrubland | 610800 | Big |
| California | Inter-Mountain Basins big sagebrush steppe | 611250 | Big |
| Great Basin | Inter-Mountain Basins big sagebrush shrubland | 1210800 | Big |
| Great Basin | Inter-Mountain Basins big sagebrush shrubland | 1310800 | Big |
| Great Basin | Inter-Mountain Basins big sagebrush shrubland | 1610800 | Big |
| Great Basin | Inter-Mountain Basins big sagebrush shrubland | 1710800 | Big |
| Great Basin | Inter-Mountain Basins big sagebrush shrubland | 1810800 | Big |
| Great Basin | Inter-Mountain Basins big sagebrush steppe | 1211250 | Big |
| Great Basin | Inter-Mountain Basins big sagebrush steppe | 1311250 | Big |
| Great Basin | Inter-Mountain Basins big sagebrush steppe | 1611250 | Big |
| Great Basin | Inter-Mountain Basins big sagebrush steppe | 1711250 | Big |
| Great Basin | Inter-Mountain Basins big sagebrush steppe | 1811250 | Big |
| Northern and Central Rockies | Inter-Mountain Basins big sagebrush shrubland | 1010800 | Big |
| Northern and Central Rockies | Inter-Mountain Basins big sagebrush shrubland | 1910800 | Big |
| Northern and Central Rockies | Inter-Mountain Basins big sagebrush shrubland | 2010800 | Big |
| Northern and Central Rockies | Inter-Mountain Basins big sagebrush shrubland | 2910800 | Big |
| Northern and Central Rockies | Inter-Mountain Basins big sagebrush shrubland - basin big sagebrush | 2110801 | Big |
| Northern and Central Rockies | Inter-Mountain Basins big sagebrush shrubland - basin big sagebrush | 2210801 | Big |
| Northern and Central Rockies | Inter-Mountain Basins big sagebrush shrubland - Wyoming big sagebrush | 2110802 | Big |
| Northern and Central Rockies | Inter-Mountain Basins big sagebrush shrubland - Wyoming big sagebrush | 2210802 | Big |
| Northern and Central Rockies | Inter-Mountain Basins big sagebrush steppe | 1011250 | Big |
| Northern and Central Rockies | Inter-Mountain Basins big sagebrush steppe | 1911250 | Big |
| Northern and Central Rockies | Inter-Mountain Basins big sagebrush steppe | 2011250 | Big |
| Northern and Central Rockies | Inter-Mountain Basins big sagebrush steppe | 2911250 | Big |
| Northern Great Plains | Inter-Mountain Basins big sagebrush shrubland | 3010800 | Big |
| Northern Great Plains | Inter-Mountain Basins big sagebrush shrubland | 3110800 | Big |
| Northern Great Plains | Inter-Mountain Basins big sagebrush steppe | 3011250 | Big |
| Northern Great Plains | Inter-Mountain Basins big sagebrush steppe | 3111250 | Big |
| Pacific Northwest | Inter-Mountain Basins big sagebrush shrubland | 110800 | Big |
| Pacific Northwest | Inter-Mountain Basins big sagebrush shrubland | 710800 | Big |
| Pacific Northwest | Inter-Mountain Basins big sagebrush shrubland | 810800 | Big |
| Pacific Northwest | Inter-Mountain Basins big sagebrush shrubland | 910800 | Big |
| Pacific Northwest | Inter-Mountain Basins big sagebrush steppe | 111250 | Big |
| Pacific Northwest | Inter-Mountain Basins big sagebrush steppe | 711250 | Big |
| Pacific Northwest | Inter-Mountain Basins big sagebrush steppe | 811250 | Big |
| Pacific Northwest | Inter-Mountain Basins big sagebrush steppe | 911250 | Big |
| Southwest | Inter-Mountain Basins big sagebrush shrubland | 1410800 | Big |
| Southwest | Inter-Mountain Basins big sagebrush shrubland | 2310800 | Big |
| Southwest | Inter-Mountain Basins big sagebrush shrubland | 2410800 | Big |
| Southwest | Inter-Mountain Basins big sagebrush steppe | 1511250 | Big |
| Southwest | Inter-Mountain Basins big sagebrush steppe | 2311250 | Big |
| Southwest | Inter-Mountain Basins big sagebrush steppe | 2411250 | Big |
| Southwest | Inter-Mountain Basins big sagebrush steppe | 2811250 | Big |
| California | Great Basin xeric mixed sagebrush shrubland | 610790 | Low |
| Great Basin | Colorado Plateau mixed low sagebrush shrubland | 1610640 | Low |
| Great Basin | Colorado Plateau mixed low sagebrush shrubland | 1710640 | Low |
| Great Basin | Columbia Plateau low sagebrush steppe | 1211240 | Low |
| Great Basin | Columbia Plateau low sagebrush steppe | 1711240 | Low |
| Great Basin | Columbia Plateau low sagebrush steppe | 1811240 | Low |
| Great Basin | Columbia Plateau scabland shrubland | 1810650 | Low |
| Great Basin | Great Basin xeric mixed sagebrush shrubland | 1210790 | Low |
| Great Basin | Great Basin xeric mixed sagebrush shrubland | 1310790 | Low |
| Great Basin | Great Basin xeric mixed sagebrush shrubland | 1610790 | Low |
| Great Basin | Great Basin xeric mixed sagebrush shrubland | 1710790 | Low |
| Great Basin | Great Basin xeric mixed sagebrush shrubland | 1810790 | Low |
| Great Basin | Inter-Mountain Basins montane sagebrush steppe - low sagebrush | 1611262 | Low |
| Great Basin | Inter-Mountain Basins semi-desert shrub-steppe | 1611270 | Low |
| Northern and Central Rockies | Columbia Plateau low sagebrush steppe | 1011240 | Low |
| Northern and Central Rockies | Columbia Plateau low sagebrush steppe | 1911240 | Low |
| Northern and Central Rockies | Columbia Plateau low sagebrush steppe | 2111240 | Low |
| Northern and Central Rockies | Columbia Plateau scabland shrubland | 1010650 | Low |
| Northern and Central Rockies | Great Basin xeric mixed sagebrush shrubland | 1010790 | Low |
| Northern and Central Rockies | Great Basin xeric mixed sagebrush shrubland | 1910790 | Low |
| Northern and Central Rockies | Great Basin xeric mixed sagebrush shrubland | 2110790 | Low |
| Northern and Central Rockies | Wyoming Basins dwarf sagebrush shrubland and steppe | 2210720 | Low |
| Northern and Central Rockies | Wyoming Basins dwarf sagebrush shrubland and steppe | 2910720 | Low |
| Pacific Northwest | Columbia Plateau low sagebrush steppe | 111240 | Low |
| Pacific Northwest | Columbia Plateau low sagebrush steppe | 711240 | Low |
| Pacific Northwest | Columbia Plateau low sagebrush steppe | 811240 | Low |
| Pacific Northwest | Columbia Plateau low sagebrush steppe | 911240 | Low |
| Pacific Northwest | Columbia Plateau scabland shrubland | 110650 | Low |
| Pacific Northwest | Columbia Plateau scabland shrubland | 710650 | Low |
| Pacific Northwest | Columbia Plateau scabland shrubland | 810650 | Low |
| Pacific Northwest | Columbia Plateau scabland shrubland | 910650 | Low |
| Pacific Northwest | Great Basin xeric mixed sagebrush shrubland | 910790 | Low |
| South-Central US | Colorado Plateau mixed low sagebrush shrubland | 2610640 | Low |
| South-Central US | Colorado Plateau mixed low sagebrush shrubland | 3410640 | Low |
| South-Central US | Inter-Mountain Basins semi-desert shrub-steppe | 2611270 | Low |
| Southwest | Colorado Plateau mixed low sagebrush shrubland | 1510640 | Low |
| Southwest | Colorado Plateau mixed low sagebrush shrubland | 2310640 | Low |
| Southwest | Colorado Plateau mixed low sagebrush shrubland | 2410640 | Low |
| Southwest | Colorado Plateau mixed low sagebrush shrubland | 2510640 | Low |
| Southwest | Colorado Plateau mixed low sagebrush shrubland | 2710640 | Low |
| Southwest | Colorado Plateau mixed low sagebrush shrubland | 2810640 | Low |
| Southwest | Inter-Mountain Basins montane sagebrush steppe - low sagebrush | 2311262 | Low |
| Southwest | Inter-Mountain Basins montane sagebrush steppe - low sagebrush | 2411262 | Low |
| Southwest | Inter-Mountain Basins semi-desert shrub-steppe | 1511270 | Low |
| Southwest | Inter-Mountain Basins semi-desert shrub-steppe | 2311270 | Low |
| Southwest | Inter-Mountain Basins semi-desert shrub-steppe | 2411270 | Low |
| Southwest | Inter-Mountain Basins semi-desert shrub-steppe | 2511270 | Low |
| Southwest | Inter-Mountain Basins semi-desert shrub-steppe | 2811270 | Low |
| Southwest | Wyoming Basins dwarf sagebrush shrubland and steppe | 2810720 | Low |

**S6: sage-grouse model posterior distributions for mapping pre-fire nest selection and survival**

The full posterior distributions for model habitat coefficients from models of sage-grouse nest habitat selection and nest survival (e.g., also described in S3 and S4) are summarized in this section by plots of their approximate probability density functions. Habitat predictors are described as either % cover, density, average value within a selected moving window size (see S1 and S2), or proximity to feature based on exponential decay functions (see S1 and S2).

**
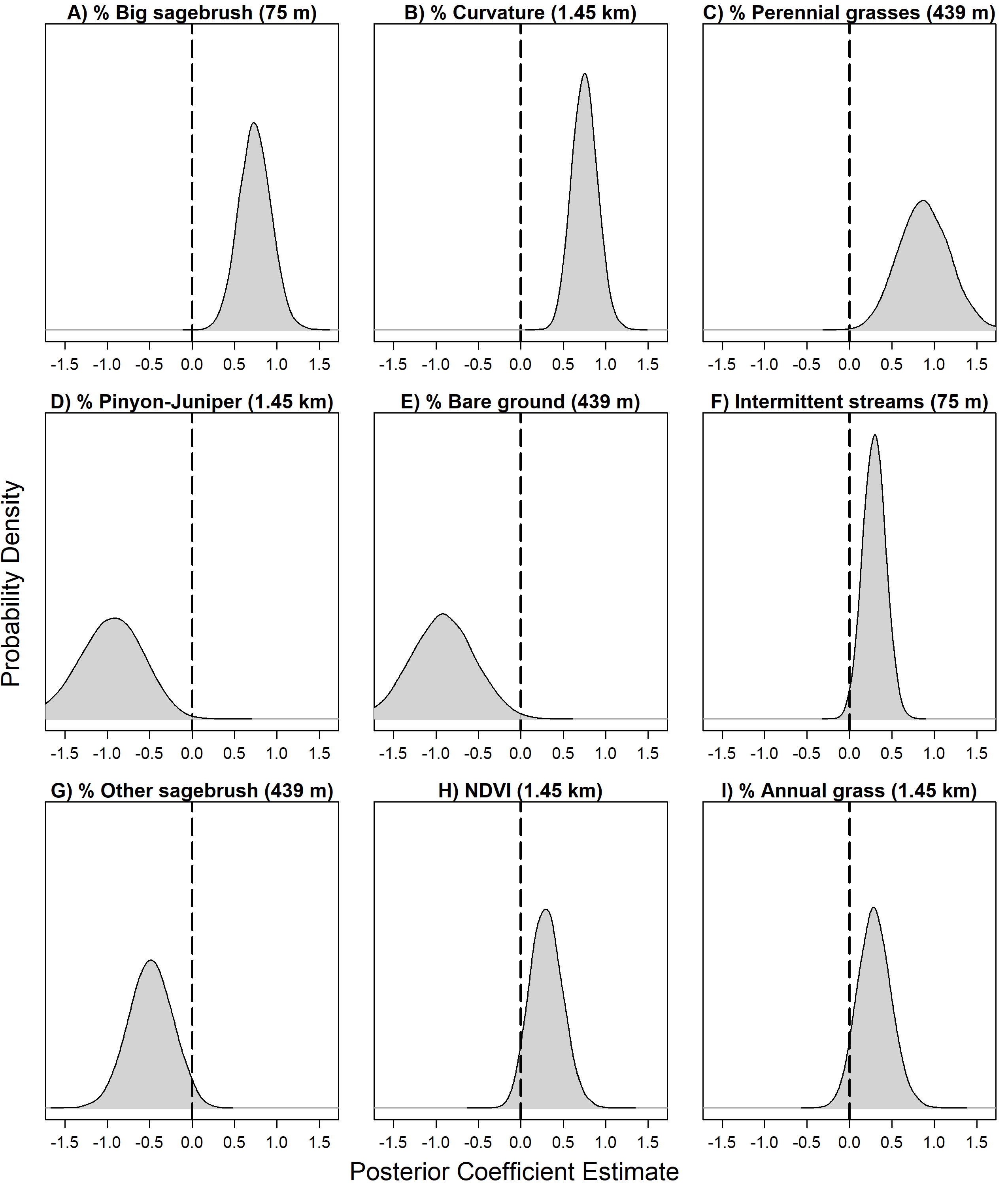
Figure S6-1.** Posterior distributions (probability density functions) of habitat coefficients from a model of nest site selection (nest resource selection function, or nest RSF) in the Virginia Mountains region of Nevada, USA. Sage-grouse location data used to build models were specific to the breeding seasons of study years 2009–2016, prior to significant wildfire events that occurred during the summers of 2016 and 2017. Values in parentheses following labels indicate the most informative radius size of each habitat predictors’ circular moving window. The dashed vertical line indicates no effect.

**
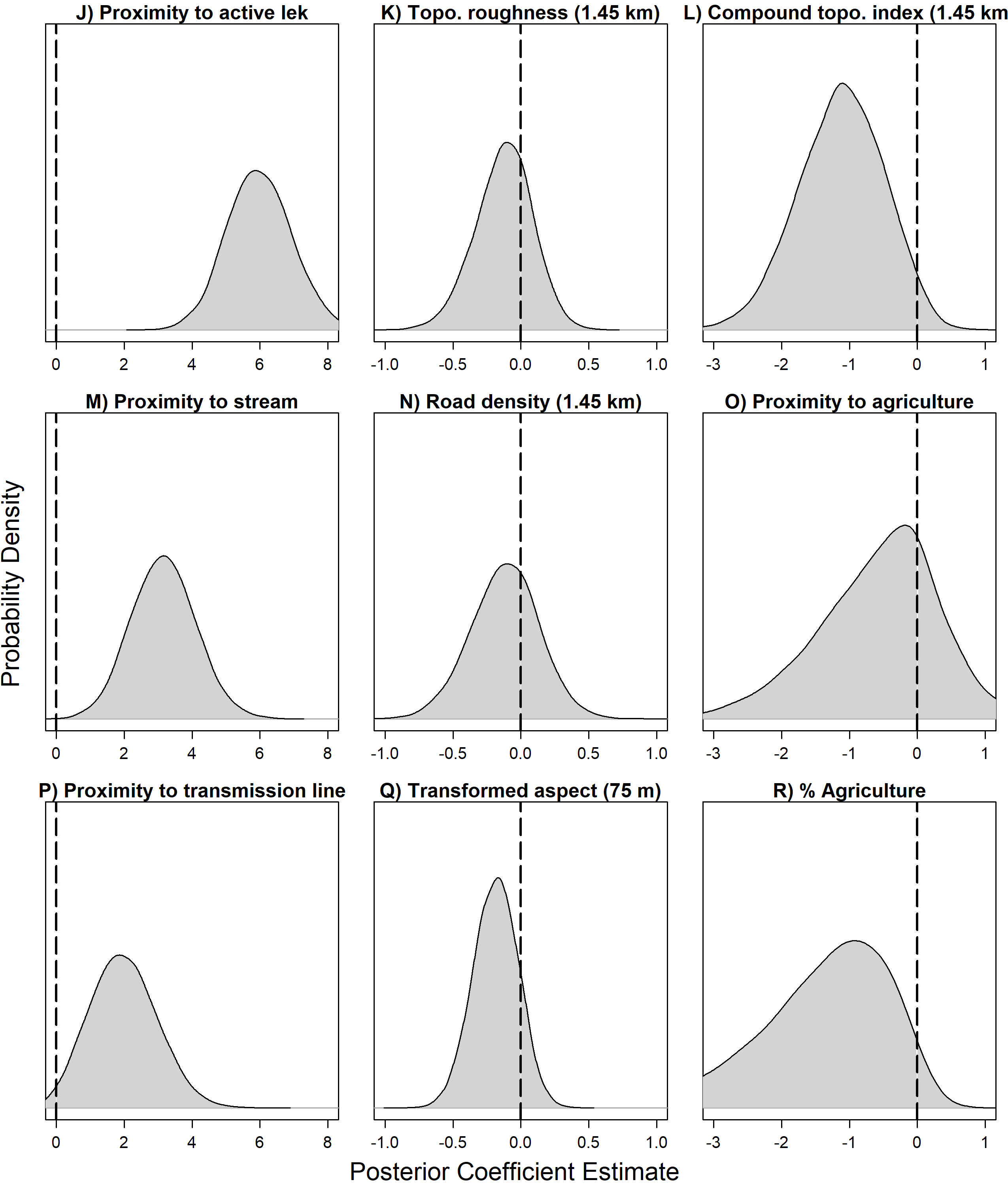
Figure S6-1, continued**

**
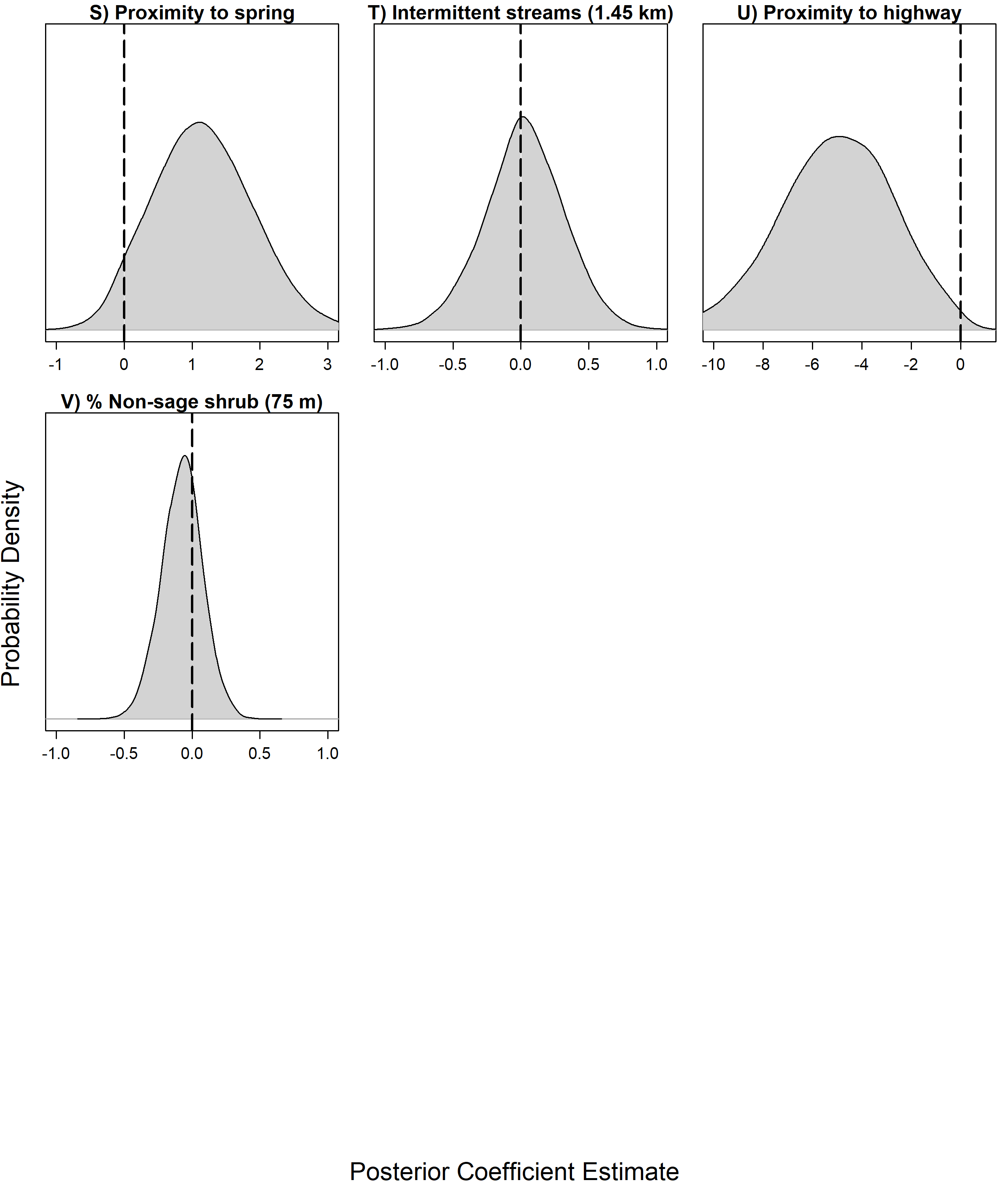
Figure S6-1, continued**

**
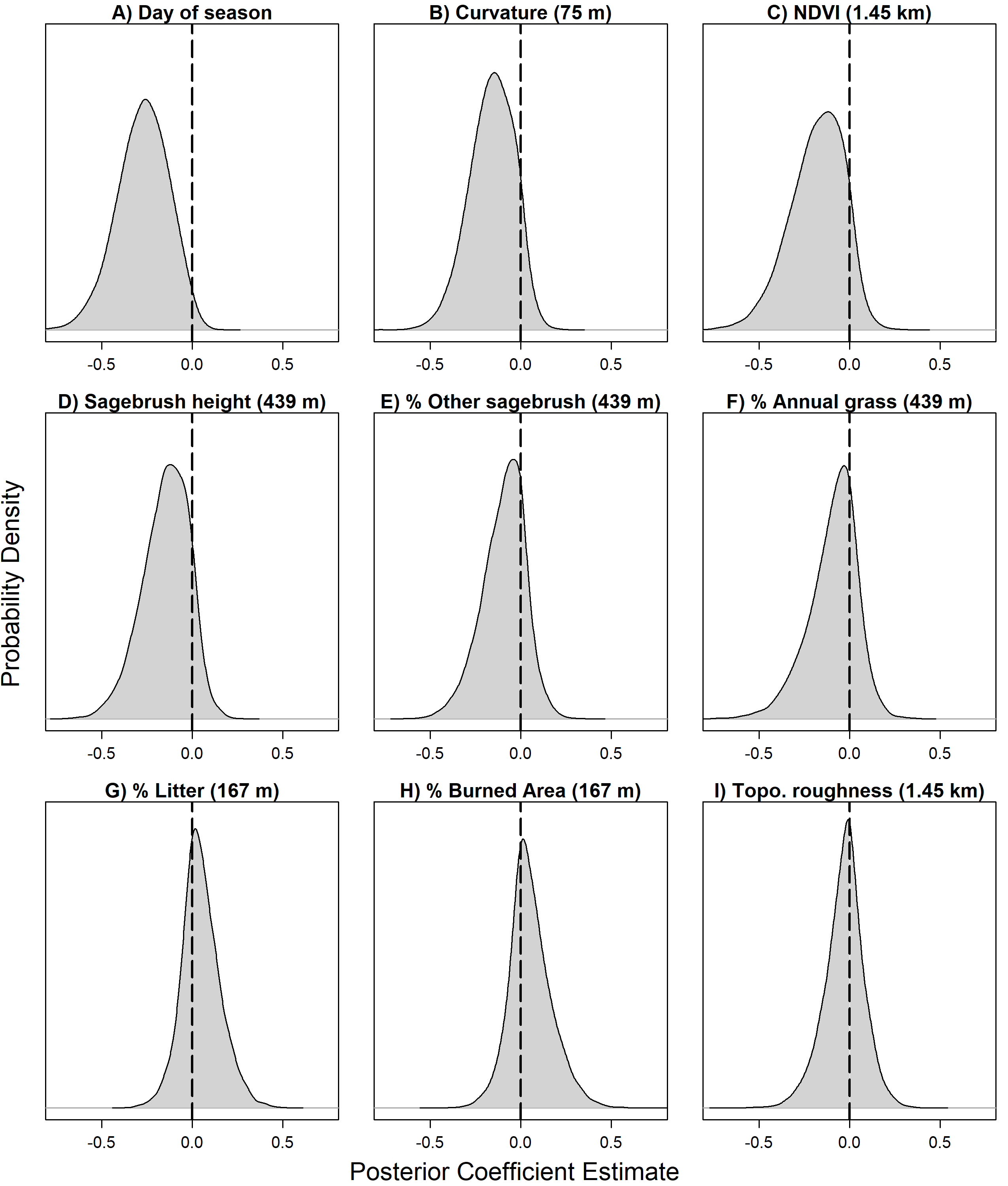
 Figure S6-2.** Posterior distributions (probability density functions) of coefficients from a hierarchical model of nest survival (nest frailty) in the Virginia Mountains region of Nevada, USA. Sage-grouse nest survival data were specific to the breeding seasons of study years 2009–2016, prior to significant wildfire events that occurred during the summers of 2016 and 2017. Values in parentheses following labels indicate the most informative radius size of each habitat predictors’ circular moving window. The dashed vertical line indicates no effect.
